# *APC* mutations dysregulate alternative polyadenylation in cancer

**DOI:** 10.1186/s13059-024-03406-4

**Published:** 2024-10-07

**Authors:** Austin M. Gabel, Andrea E. Belleville, James D. Thomas, Jose Mario Bello Pineda, Robert K. Bradley

**Affiliations:** 1 Computational Biology Program, Public Health Sciences Division, Fred Hutchinson Cancer Center, Seattle, WA USA; 2 Basic Sciences Division, Fred Hutchinson Cancer Center, Seattle, WA USA; 3 Department of Genome Sciences, University of Washington, Seattle, WA USA; 4 Medical Scientist Training Program, University of Washington, Seattle, WA USA; 5 Molecular and Cellular Biology Program, University of Washington, Seattle, WA USA

## Abstract

**Background:**

Alternative polyadenylation (APA) affects most human genes and is recurrently dysregulated in all studied cancers. However, the mechanistic origins of this dysregulation are incompletely understood.

**Results:**

We describe an unbiased analysis of molecular regulators of poly(A) site selection across The Cancer Genome Atlas and identify that colorectal adenocarcinoma is an outlier relative to all other cancer subtypes. This distinction arises from the frequent presence of loss-of-function *APC* mutations in colorectal adenocarcinoma, which are strongly associated with long 3′ UTR expression relative to tumors lacking *APC* mutations. *APC* knockout similarly dysregulates APA in human colon organoids. By mining previously published APC eCLIP data, we show that APC preferentially binds G- and C-rich motifs just upstream of proximal poly(A) sites. Lastly, we find that reduced *APC* expression is associated with APA dysregulation in tumor types lacking recurrent *APC* mutations.

**Conclusions:**

As APC has been previously identified as an RNA-binding protein that preferentially binds 3′ UTRs during mouse neurogenesis, our results suggest that APC promotes proximal poly(A) site use and that APC loss and altered expression contribute to pervasive APA dysregulation in cancers.

**Supplementary Information:**

The online version contains supplementary material available at 10.1186/s13059-024-03406-4.

## Background

Alternative cleavage and polyadenylation (APA) occurs in a majority of human genes and is known to vary across cell types and biological contexts, including cancer [1–6]. Mechanistic study of distinct APA events demonstrates that changes in poly(A) site selection can alter mRNA stability, translation kinetics, and localization [7–9]. APA is globally dysregulated in all studied human cancers [10, 11], and alterations in APA correlate with clinically relevant cancer phenotypes such as patient prognosis, immune infiltration, and others [10, 12–14].

Although canonical protein regulators of APA and 3′ end processing of mRNAs have been well studied and demonstrated to contribute to cancer-associated dysregulation of APA, the mechanistic origins of the widespread APA dysregulation that characterizes almost all cancers remains incompletely understood. Previous analyses of tumor and matched normal control samples have identified *CSTF2* (*CstF64*) as a potential pivotal regulator of 3′ UTR shortening across a subset of human cancers [11]. Interestingly, expression of other core components of the 3′ end processing complex, including *NUDT21* and *PCF11*, which both have compelling evidence for a key role in poly(A) site selection from targeted knockdown experiments [15, 16] did not display strong associations with global changes in APA across tumor types [11]. Other studies have assessed correlations between gene expression of putative regulators of poly(A) site selection and APA, focusing on genes previously associated with the 3′ end processing complex [10, 11, 16]. The potential contributions of other genes, including those that are not historically thought of as core components of the APA machinery, to modulation of poly(A) site selection in both healthy and cancerous cells likely have not yet been fully elucidated.

## Results

### Expression of canonical poly(A) regulators correlates with global polyadenylation site selection in all *cancer* subtypes except colorectal adenocarcinoma

**Fig. 1:**
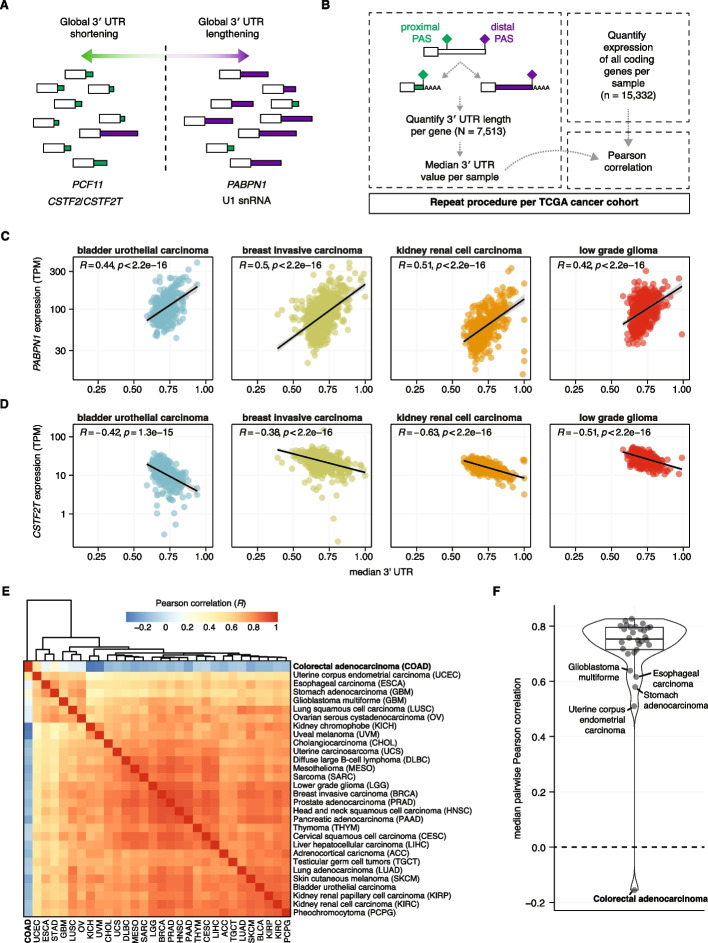
Global regulators of poly(A) site selection correlate with 3′ UTR length in all cancer subtypes except colorectal adenocarcinoma. **A** Graphical summary of poly(A) site modulators and how they are purported to act globally based on computational correlations with 3′ UTR measurements and targeted knockdown experiments. **B** Workflow to assess global correlations of gene expression and a summary statistic of 3′ UTR length. For each sample, the 3′ UTR length is calculated per gene using previously published measurements using DaPars [11] for all TCGA data. For each sample, a summary statistic of 3′ UTR length was calculated by taking the median of all imputed 3′ UTR lengths, referred to as median 3′ UTR. We then quantify gene expression (transcripts per million, TPM) for all coding genes per RNA-seq sample across the TCGA RNA-seq datasets. Then for each gene a Pearson correlation was completed comparing median 3′ UTR length and gene expression per gene, per dataset. **C** Scatter plot of 3′ UTR length and *PABPN1* expression (TPM) for four TCGA datasets. Each point represents a single RNA-seq sample. *R* and *p* value reflective of the Pearson correlation. **D** Scatter plot of 3′ UTR length and *CSTF2T* expression (TPM) for four TCGA datasets. Each point represents a single RNA-seq sample. *R* and *p* value reflective of the Pearson correlation. **E** Correlation matrix of all 30 datasets comparing calculated Pearson correlation coefficients comparing gene expression of each individual gene versus median 3′ UTR. Pairwise dataset correlations were calculated using Pearson correlation. **F** Violin plot of the median pairwise Pearson correlation comparing all analyzed gene expression—3′ UTR correlations obtained for each cohort to one another

To identify potential regulators of APA in human cancer, we assessed the correlation between gene expression and a summary statistic of 3′ UTR across RNA-seq datasets of 29 distinct cancer subtypes from The Cancer Genome Atlas (TCGA). Our approach builds off of previous studies which focused on smaller subsets of tumors for which peritumoral, healthy control tissues are available (Fig. 1A) [10, 11]. For each RNA-seq sample within a given dataset, we imputed the 3′ UTR length per gene using existing computational tools, in this case, DaPars [11]. We then took the median of all imputed gene level 3′ UTR measurements to generate a median 3′ UTR per sample that serves as a summary statistic of global poly(A) site selection [17]. A larger value indicates a given sample uses, on average, more distal poly(A) sites, and thus the 3′ UTR of any given gene tends to be longer. We next calculated gene expression of all coding genes present in our lab annotation (*N* = 15,332) represented as transcripts per million (TPM). We then correlated the median 3′ UTR and gene expression (TPM) across a given dataset and obtained a Pearson correlation coefficient per gene within each cancer type (Fig. 1B). A positive Pearson correlation coefficient indicates higher expression of that gene is associated with, on average, longer 3′ UTRs in that cancer type, and a negative–positive Pearson correlation coefficient indicates the opposite.

We first sought to validate this approach for genes known to control poly(A) site selection globally. This includes *PABPN1*, where high levels are known to reduce proximal poly(A) site selection [16, 18], and the paralogs *CSTF2* and *CSTF2T*, where high expression correlates with increased use of proximal poly(A) site selection [10, 11, 19], and thus should have either a positive or negative correlation using our approach. We applied this method to RNA-seq data from tumors across 30 cancer subtypes in the Cancer Genome Atlas (TCGA) database and quantified gene expression—median 3′ UTR correlations for each cancer subtype.

As expected, we observed *PABPN1* and *CSTF2T* expression are positively and negatively correlated with median 3′ UTR length, respectively (Fig. 1C–D; Additional file 1: Fig. S1A–C). In addition, we identified strong correlations with other genes previously identified as core components of the mRNA 3′ end processing machinery including *CSTF3*, *CPSF2*, and *NUDT21* (Additional file 1: Fig. S1D; Additional file 2–3: Table S1–S2). We next sought to understand how similar correlations of gene expression and 3′ UTR length were between distinct cancer subtypes. Pairwise comparisons of subtype correlations of gene expression and median 3′ UTR length were remarkably similar across most TCGA cancer subtypes analyzed, with a median pairwise Pearson correlation coefficient of 0.699 (Fig. 1E–F). Despite the concordance across most datasets, colorectal adenocarcinoma stood out as a striking outlier (median pairwise Pearson correlation coefficient of − 0.14), and known regulators of poly(A) site selection such as *PABPN1* and *CSTF2T* showed no correlation with median 3′ UTR length, unlike in all other analyzed datasets (Fig. 1E–F; Additional file 1: Fig. S1A–D). This suggested there may be some unique feature of colorectal adenocarcinoma that obscures the expected correlations with known regulators of poly(A) site selection, perhaps by altering poly(A) site selection in a manner that is distinct from all other analyzed cancer subtypes.

### *APC* nonsense and frameshift mutations are associated with enhanced distal poly(A) site selection in colorectal adenocarcinoma

Colorectal adenocarcinomas are largely driven by mutations in the gene adenomatous polyposis coli (*APC*) [20], and inherited loss-of-function mutations in *APC* cause familial adenomatous polyposis, which carries nearly a 100% risk of colorectal adenocarcinoma development at some point in life [21–24]. Most studies of APC in colorectal adenocarcinoma focus on the protein’s role in regulating WNT signaling or cytoskeletal organization; however, several studies focused on murine neurogenesis have previously identified APC as an RNA-binding protein [25–27]. Crosslinking immunoprecipitation sequencing (CLIP-seq) of APC–RNA binding revealed more than 90% of identified APC binding sites are in the 3′ UTR of the target mRNA [26]. This identified role of APC as an RNA-binding protein has been largely unexplored in the context of cancer, and a link between APC and global regulation of 3′ UTR length is unreported in any context.

**Fig. 2:**
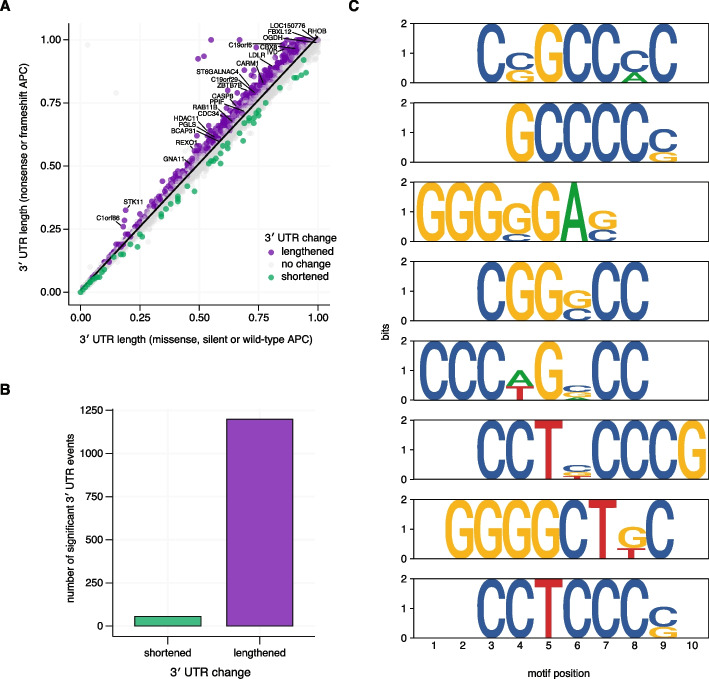
APC nonsense and frameshift mutations are associated with enhanced distal poly(A) site use in colorectal adenocarcinoma. **A** Scatter plot of the median imputed 3′ UTR length using DaPars for samples without APC loss-of-function mutations versus samples with a nonsense or frameshift APC mutation. All 3′ UTR measurements per group were compared using a two-sided Wilcoxon rank-sum test. Comparisons with a Benjamin-Hochberg corrected* p* value < 0.05 were called as significantly altered. Points labeled in green indicate significant shortening in APC nonsense or frameshift samples, points labeled in purple indicate significant lengthening in APC nonsense or frameshift samples, and points labeled in gray indicate no significant difference. **B** Bar plot of significantly altered lengthening (purple, n = 1198) or shortening events (green, *n* = 55) in APC nonsense or frameshift samples. **C** Sequence logo plot of de novo motif enrichment of 3′ UTRs exhibiting significant lengthening in APC nonsense or frameshift colorectal adenocarcinoma samples compared to all other UTRs analyzed in panel A that exhibit no significant change in 3′ UTR length. Top 8 significantly enriched motifs displayed

We first reasoned that frameshift or nonsense mutations in APC were more likely to disrupt RNA binding capacity, as opposed to missense mutations, which may have more heterogeneous effects on protein function. We stratified colorectal adenocarcinoma samples into two groups that either harbored at least one nonsense or frameshift mutation (*N* = 342) or those without a nonsense or frameshift mutation (*N* = 282). Samples with a frameshift or nonsense mutation displayed significant, global 3′ UTR lengthening compared to samples without a frameshift or nonsense mutation (Fig. 2A–B; Additional file 4: Table S3). We validated this result with two distinct algorithms used to quantify 3′ UTR length—DaPars and APAlyzer—which use distinct computational approaches to quantify 3′ UTR length (Additional file 1: Fig. S2A–E) [11, 28]. While this approach has limitations, specifically that some missense mutations may also result in protein loss-of-function, we validated the stratification by completing differential gene expression analysis, which revealed that samples with at least one nonsense or frameshift mutation expressed significantly higher levels of canonical WNT signaling genes as expected [20] (Additional file 1: Fig. S3A–C).

We then completed de novo motif enrichment of lengthened 3′ UTRs to computationally predict potential APC binding sites [29] and found that the top 8 motifs were largely G- and C-rich (Fig. 2C). This is concordant with results from a prior study of mouse APC CLIP-seq data, which identified three consensus motifs: a G-rich motif, a C-rich motif, and CUGU [26]. These data indicate APC may function directly or indirectly to promote proximal poly(A) site use in a sequence-specific manner.

### Targeted APC knockout in human *colon* organoids alters poly(A) site selection

**Fig. 3:**
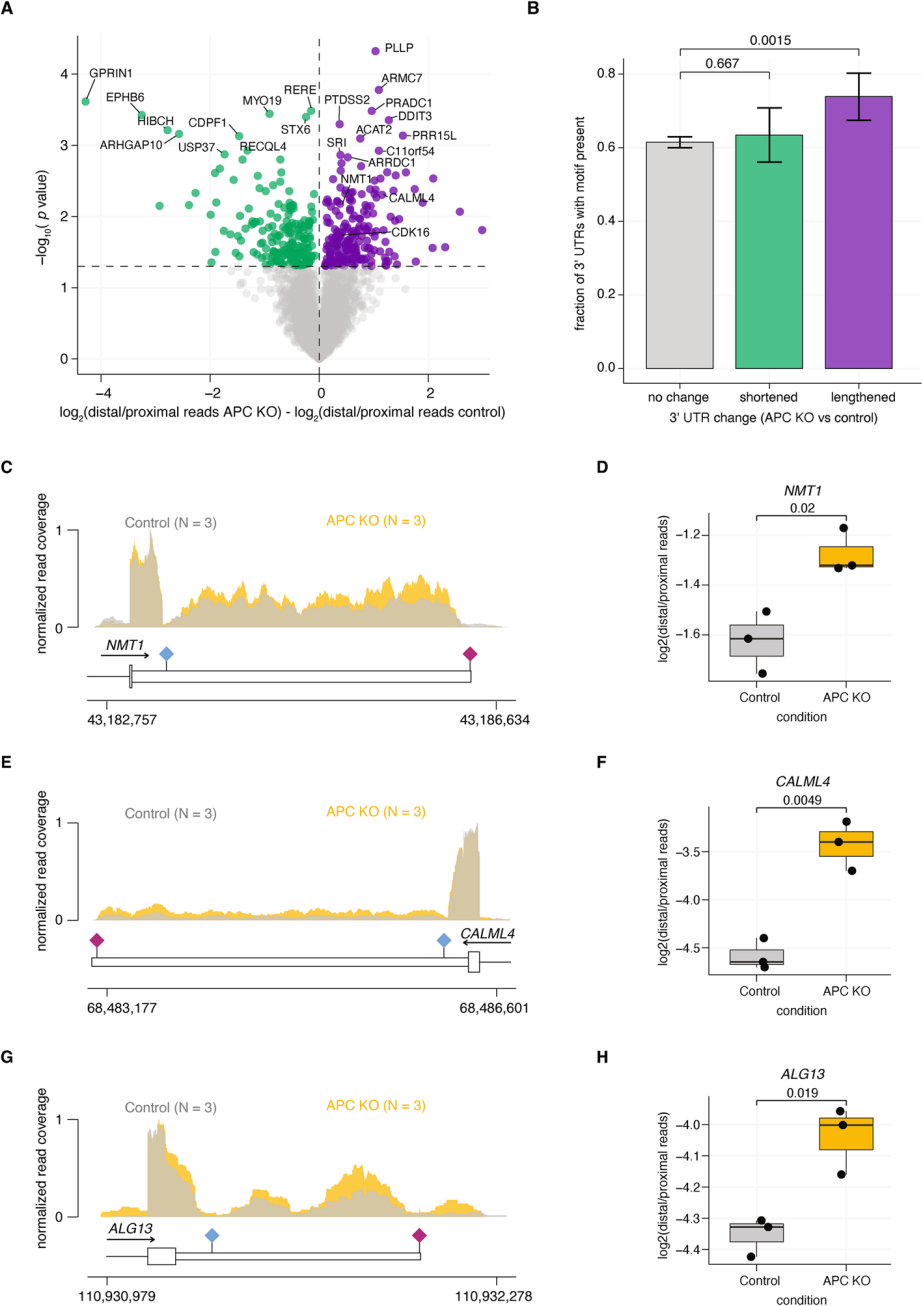
Acute induction of APC loss in colon organoids drives global dysregulation of poly(A) site selection. **A** Scatter plot of the difference in gene level 3′ UTR lengths in human colon organoids with APC gene knockout using CRISPR/Cas9 comparing 0- versus 24-h timepoints. 3′ UTRs that show significant shortening are indicated in green lengthening are indicated in purple. **B** Fraction of 3′ UTRs identified in colon organoids as lengthened, shortened, or no change (panel **A**) that contain at least one of the five most significantly enriched motifs (CSGGCCMC, GCCCCS, GGGGGAS, CGGSCC, CCCWGSCC) identified from colorectal adenocarcinoma de novo motif enrichment analysis (Fig. 2D). *P* values were calculated using a Chi-squared test compared to the 3′ UTRs in the no change group; error bars reflect 95% confidence interval using bootstrapping. **C**,** E**,** G** Bam coverage plots of RNA-seq data for individual 3′ UTRs (*NMT1*, *CALML4*, *ALG13*) that display lengthening 24 h after APC knock out. Plots are overlaid for time 0 (gray, *n* = 3) or 24 h (yellow, *n* = 3). **D**,** F**,** H** Boxplots of 3′ UTR length quantified per gene using APAlyzer, values reflect log_2_(distal/proximal reads). *P* value reflective of a two-sided Student’s *T*-test

As RNA-seq obtained from cancer samples are often extremely heterogeneous in terms of cell populations, genetic background, and sample quality, we wanted to assess alterations in APA in a more controlled experimental setting. We analyzed previously published data from human colon organoids at baseline or 24 h after targeted knockout of *APC* using CRISPR/Cas9 [30]. We computed differentially polyadenylated 3′ UTRs and identified 196 shortened and 207 lengthened 3′ UTRs 24 h after *APC* knockout (Fig. 3A; Additional file 5: Table S4).

We screened all analyzed 3′ UTRs in the organoid data for presence of at least one of the top five enriched motifs from COAD (CSGGCCMC, GCCCCS, GGGGGAS, CGGSCC, CCCWGSCC) and found that lengthened 3′ UTRs, but not shortened 3′ UTRs, were significantly more likely to contain at least one of these motifs (73.8%) compared to unchanged 3′ UTRs (61.4%) (Fig. 3B). We validated 3′ UTR lengthening events by visualizing the RNA-seq data as overlaid BAM coverage plots and comparing the quantified 3′ UTR length per condition for a number of genes (Fig. 3C–H). These data demonstrate that APC loss-of-function likely perturbs poly(A) site selection leading to 3′ UTR lengthening of a subset of 3′ UTRs enriched in G- and C-rich motifs.

### APC binds mRNAs proximal to canonical poly(A) sites

**Fig. 4:**
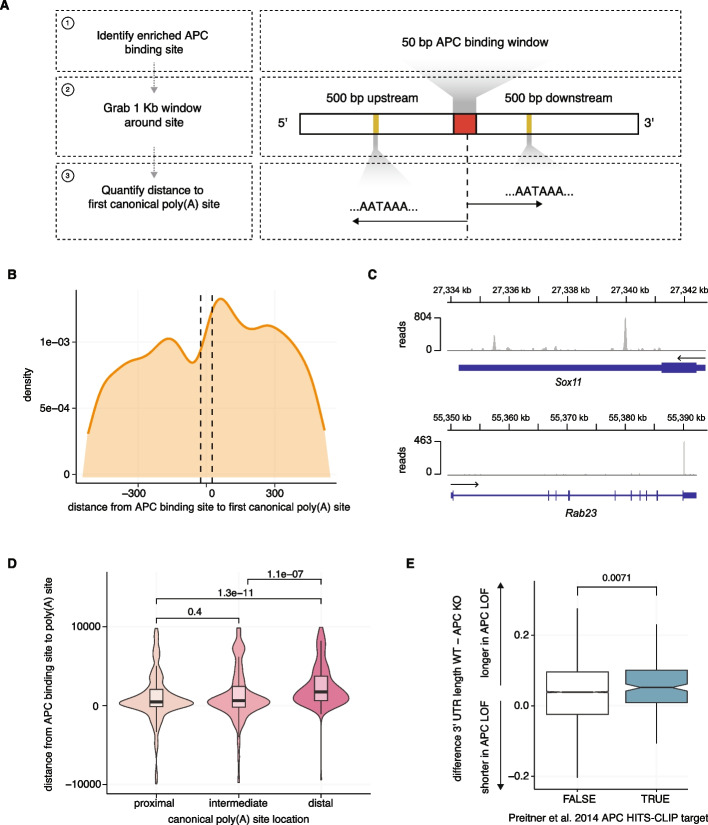
APC binds mRNAs proximal to poly(A) signal sequences. **A** Overview of computational pipeline, including mapping of HITS-CLIP data and detection of enriched binding sites using DEWseq, definition of a 1000-bp widnow surrounding identified binding sites, and quantification of distance to the nearest canonical poly(A) signal sequences (AATAAA or ATTAAA). **B** Density plot of nearest canonical poly(A) sequence from identified APC binding sites using HITS-CLIP. Dotted lines indicate a 50-bp window of identified HITS-CLIP binding. Maximal signal occurring at + 62.19 bp from the center of APC binding. **C** Representative read coverage plots of APC HITS-CLIP binding data. Plots illustrate two of the most-enriched genes, *Sox11* and *Rab23*, identified from reanalysis of the APC HITS-CLIP binding data from Preitner et al. (2014) [26]. **D** Violin plots of distance from APC binding site to high-confidence poly(A) sites from PolyADB v3 [32]. Poly(A) sites are binned into either proximal (most 5′ poly(A) signal within the 3′ UTR), distal (most 3′ poly(A) signal within the 3′ UTR), or intermediate (all other poly(A) sites). *P* values from a two-sided Wilcoxon rank-sum test. **E** Boxplot comparing the differences in change of 3′ UTR length between colorectal adenocarcinoma samples with or without *APC* loss-of-function mutations for genes identified as direct binding targets of APC from Pretiner et al. (2014) (blue) or genes not identified in their experiments (white). A more positive value indicates, on average, a longer 3′ UTR in the *APC* loss-of-function colorectal adenocarcinoma samples. *P* value from a two-sided Wilcoxon rank-sum test

To gain further insight into the relationship between APC RNA binding and poly(A) site selection, we reanalyzed previously published APC HITS-CLIP data (Fig. 4A) [26]. We identified enriched APC binding sites in the CLIP data and searched a 1000-bp window around those sites to evaluate the distance to canonical poly(A) signal sequences (AATAAA,ATTAAA) [3, 31]. We found that APC binding sites exhibited enrichment of canonical poly(A) sequences proximal to APC binding sites, with a maximum density of poly(A) signal sequences ~ 62 bp downstream of APC binding sites (Fig. 4B; Additional file 1: Fig. S4A; Additional file 6: Table S5). We then augmented those analyses by testing for association between APC binding and APA. We assessed where APC binds RNAs relative to proximal versus distal poly(A) sites within the 3′ UTR. We used a published database of annotated poly(A) signal sequences [32] and measured the distance between APC binding sites and annotated poly(A) signal sequences to find that APC binds significantly closer to proximal and intermediate poly(A) signal sequences than it does to distal poly(A) signals (Fig. 4C–D). These analyses indicate that APC typically binds mRNAs immediately upstream of more proximal canonical poly(A) sequences within 3′ UTRs.

Given the similarity in identified motifs between our computational analyses of human transcriptomes and experimental measurements in mice (Fig. 2C) [26], we reasoned the identified RNA targets of APC binding may demonstrate more significant phenotypes than do other genes in our analysis. We assessed the degree of 3′ UTR lengthening among APC-bound genes identified from HITS-CLIP data and found that those genes, on average, displayed significantly more lengthening than did other genes included in our analysis not identified as APC targets in Preitner et al. (Fig. 4E). When assessing the fraction of the APC binding targets displaying significant lengthening, we found that the observed fraction of genes displaying significant lengthening (17.6%) was significantly higher than what we would expect to observe based on random chance (Additional file 1: Fig. S4B–E). Taken together, these data demonstrate that APC binds RNA proximal to canonical poly(A) signal sequences and suggest that the RNA-binding targets identified in mice are potentially conserved in humans.

### *APC* expression correlates with 3′ UTR length and expression levels of genes involved in growth factor signaling in low-grade glioma

**Fig. 5:**
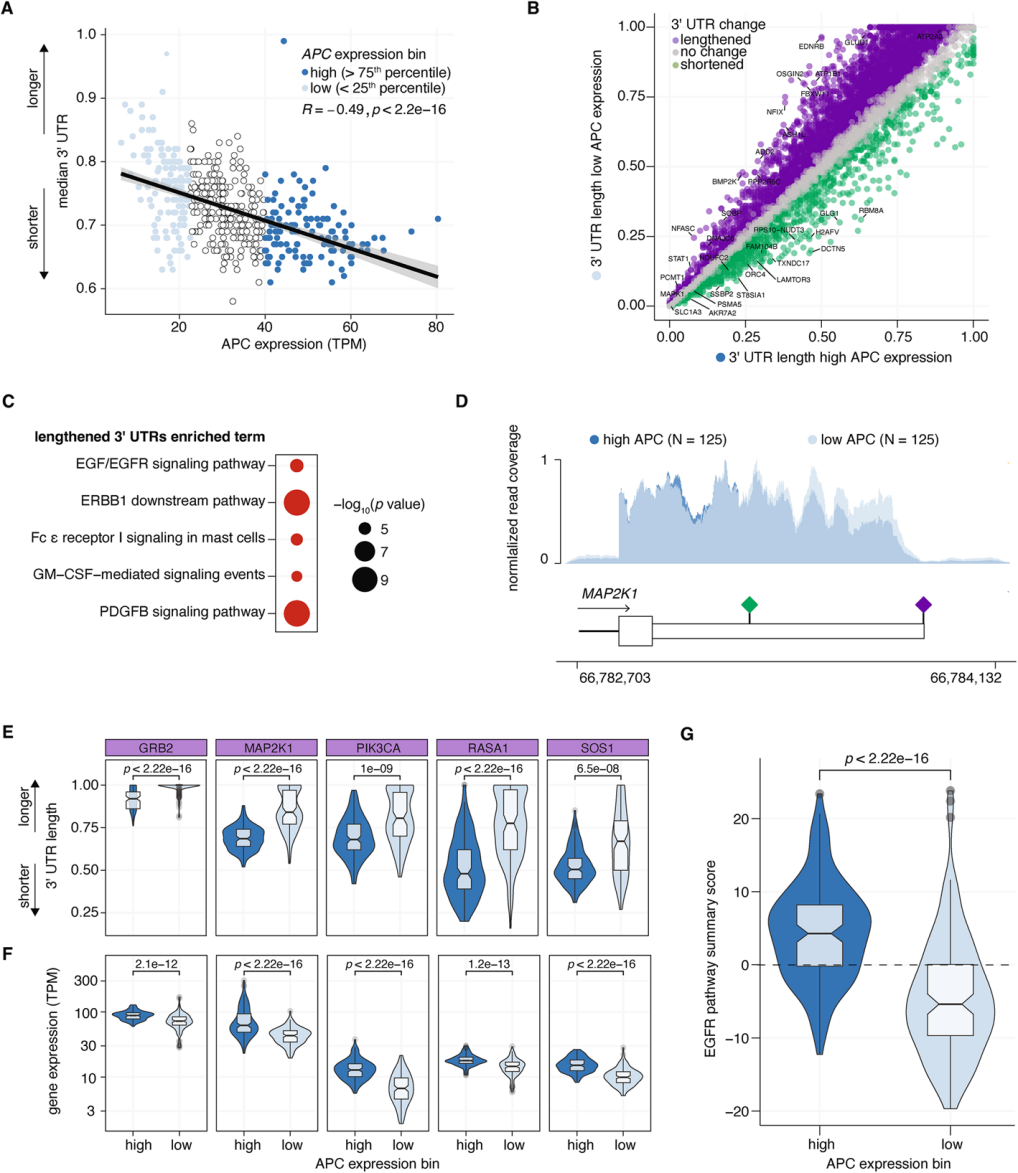
Low APC expression correlates with 3′ UTR lengthening in low-grade glioma. **A** Scatter plot of median 3′ UTR versus APC gene expression (TPM) per sample in the TCGA low-grade glioma cohort (*N* = 500). Samples are colored by expression bin. P value and R from Pearson correlation. **B** Differential poly(A) site use in high versus low APC expressing low-grade glioma samples. Genes with 3′ UTR lengthening are indicated in purple, no change in gray, and shortening in green. **C** Top 5 enriched pathways of lengthened 3′ UTRs in APC low expressing low-grade glioma samples. **D** BAM coverage plot of RNA-seq of MAP2K1 3′ UTR from low-grade glioma samples stratified by APC expression bin (*N* = 125 each bin). Schematic of MAP2K1 3′ UTR indicates the proximal poly(A) sequence with a green diamond and the distal poly(A) sequence with a purple diamond. **E** Violin plot of 3′ UTR length of several EGFR signaling genes in high versus low APC expressing stratified low-grade glioma samples. *P* value from two-sided Wilcoxon rank-sum test. **F** Violin plot of gene expression of several EGFR signaling genes in high versus low APC expressing stratified low-grade glioma samples. *P* value from two-sided Wilcoxon rank-sum test. **G** Violin plots of EGFR signaling pathway summary score (summed *Z* score of gene expression across 28 genes) per sample for low-grade glioma samples stratified by APC expression bin. *P* value from two-sided Wilcoxon rank-sum test

To evaluate if these analyses were relevant in other contexts, we assessed global correlations of poly(A) site selection and *APC* gene expression in other cancer subtypes. Brain tissues generally demonstrate the highest expression of *APC* [33] and, concordantly, low-grade gliomas display the highest expression of *APC* among all TCGA cancer subtypes (Additional file 1: Fig. S5A). We first analyzed the correlation between *APC* expression and global median 3′ UTR length and found that higher *APC* expression was associated with significantly shortened 3′ UTRs globally (Fig. 5A). We next stratified patients into high (> 75th percentile) and low (< 25th percentile) *APC* expression to identify differentially polyadenylated genes, which revealed 5517 lengthening and 1158 shortening events in *APC* low expression samples (Fig. 5B; Additional file 7: Table S6). Gene ontology (GO) analysis of lengthened 3′ UTRs revealed significant enrichment for genes involved in a number of growth factor signaling pathways, including EGFR and PDGF signaling cascades (Fig. 5C). EGFR signaling is clinically relevant and therapeutically targetable in low-grade gliomas as well as other brain and non-brain malignancies [34], and so we sought to further characterize APA of genes involved in EGFR signaling pathways.

We confirmed 3′ UTR lengthening of several genes involved in EGFR signaling cascades that corresponded with significant reductions in gene expression that clustered by *APC* expression (Fig. 5D–F). Of 28 genes identified as hallmark EGFR signaling cascade genes, 21 demonstrated significant 3′ UTR lengthening, 7 displayed no change and 1 displayed significant shortening in *APC* low expression low-grade glioma samples (Additional file 1: Fig. S5B–E). We next devised an EGFR summary score, defined as the sum of *Z* scores of gene expression across 28 genes per sample, and found that APC low expressing samples demonstrated significantly reduced EGFR signaling scores compared to APC high expression samples (Fig. 5G). These data demonstrate that APC expression levels correlate with global alterations in poly(A) site selection, which are associated with significant alterations in gene expression of a clinically actionable signaling pathway.

## Discussion

Here, we build on previous insights that APC is an RNA-binding protein that binds overwhelmingly in the 3′ UTR during mouse neurogenesis [26]. We extend those findings to human cancers and show that *APC* loss-of-function mutations as well as reduced expression are strongly correlated with 3′ UTR lengthening for many RNA targets. Among these targets are WNT signaling genes, as demonstrated by changes in 3′ UTR length in low-grade glioma (Fig. 5C–G). Differential expression of APC and corresponding altered poly(A) site selection could thereby influence gene expression of key growth-promoting pathways, including WNT signaling genes, that are implicated in tumorigenesis. Such a hypothesis is concordant with observations from colorectal adenocarcinoma, where homozygous deletions of *APC* are extremely rare and thought to arise from a need to optimize β-catenin activity [35]. The functional and potentially pro-tumorigenic consequences of the 3′ UTR lengthening associated with *APC* loss-of-function mutations or reduced expression remains unclear—in particular, it is unknown which specific 3′ UTR lengthening events are relevant to cancer progression—but this important question could be experimentally answered by functionally interrogating poly(A) site selection in a multiplexed fashion in relevant biological models [17].

Our study motivates several avenues of investigation for future work. First, targeted knockdown of *APC* coupled with sequencing methods developed to unambiguously map alterations in poly(A) site selection such as Poly(A)-seq could refine and focus subsequent functional study of individual genes exhibiting significantly altered 3′ UTRs. Second, further study of protein–protein and protein-RNA interactions with techniques like eCLIP or proximity labeling is needed to strengthen our analysis indicating that APC frequently binds RNA proximal to canonical poly(A) sequences (Fig. 4B). Such studies could validate this result in human cells and yield specific insights into the molecular mechanisms by which APC shapes poly(A) site selection. For example, immunoprecipitation mass spectrometry data has identified components of the U4/5/6 tri-snRNP complex as direct interactors with APC [36]. While these components are not classically linked to poly(A) site selection, direct binding of APC to mRNA as well as components of the canonical poly(A) processing or splicing machineries could directly or indirectly promote cleavage at proximal poly(A) sites. Finally, focused investigation of how the altered poly(A) site selection driven by APC loss influences tumorigenesis and tumor phenotypes may yield new insights into the process of cellular transformation. For example, rapid cell division is strongly associated with global 3′ UTR shortening [37], raising interesting questions about cellular growth kinetics following the acquisition of *APC* loss-of-function mutations.

There are several limitations to our work that are important to note. First, the association between *APC* knockdown and 3' UTR lengthening in organoids is clearly evident for transcripts containing enriched motifs identified via motif enrichment analyses in primary human cancer samples but not when this restriction is removed. We hypothesize that this may be due to the relatively short interval (24 h) between *APC* knockdown and collection for RNA-seq but cannot directly test this hypothesis without additional experimental evidence. Second, we have not directly demonstrated that APC is directly (mechanistically) responsible for changes in poly(A) site selection in human cells. Although previous studies in mouse cells demonstrated direct binding of APC to 3′ UTRs [26], we cannot exclude the hypothesis that the global shifts in APA site selection that we observe occur downstream of changes in gene expression induced by low levels of functional APC. Given this, we cannot say with certainty that *APC* mutations are solely or even primarily responsible for the distinct global patterns of APA that we observed in colorectal adenocarcinoma samples (Fig. 1E–F). Third, although we present exploratory analyses indicating that *APC* expression is correlated with alterations in APA as well as with expression of genes in pathways critical for tumor growth and survival, additional work is needed to assess the potential functional relevance of those changes to tumorigenesis. We hope that this work motivates future study to mechanistically characterize how *APC* shapes poly(A) site selection in cancer as well as functional study of specific APA events that contribute to tumorigenesis.

## Methods

### RNA-seq mapping and analysis

RNA-seq was analyzed as previously described [38]. RNA-seq reads were mapped to an annotated transcriptome created using Ensembl 71 [39] UCSC knownGene [40] and Misov2.0 [41] annotations using RSEM version 1.2.4 [42] (modified to call Bowtie [43] with option “-v 2”). Unaligned reads were then mapped to the human genome (hg19/GRCh37 assembly) and a database containing all possible pairings of 5′ and 3′ splice sites per gene in our merged transcriptome annotation using TopHat version 20.8b [44]. All mapped reads were merged and then input into MISO v2.0. For TCGA studies, we analyzed 9045 available samples across 30 cancer types.

## 3’ UTR analyses

For TCGA data, gene level 3′ UTR measurements were downloaded from a previously published database [45] or BAM files were analyzed using the APAlyzer package in R [28] and the gene level log_2_(distal reads/proximal reads) was computed per sample. For the Schwank 2020 organoid data, RNA-seq data was mapped as stated above and BAM files were analyzed using the APAlyzer package in R [28] and the gene level log_2_(distal reads/proximal reads) was computed per sample for each respective strand.

## Median 3’ UTR analyses and Pearson correlation

For each TCGA sample, we computed the median 3′ UTR of all assayed genes, referred to as the median 3′ UTR. We then assessed the Pearson correlation between gene expression (transcripts per million) computed as described above and the median 3′ UTR among each TCGA cancer subtype for every gene in our lab annotation (*N* = 15,332). We then assessed the Pearson correlation coefficient *R* and the *P* value for each gene expression and median 3′ UTR correlation with Bonferroni correction for multiple hypothesis testing.

We then completed pairwise Pearson correlation analysis of all the distinct Pearson correlations obtained for each TCGA cancer subtype. We calculated the median Pairwise Pearson correlation between the datasets by assessing every calculated Pearson correlation between each TCGA subtype and then taking the median value.

All statistical analyses were performed in R with Bioconductor, and the tables and plots were generated using dplyr [46] and ggplot2 [47] packages.

## APC mutation calling

Mutation Annotation Files (MAF) files for the TCGA colorectal adenocarcinoma dataset were downloaded from the NCI Genomic Data Consortium (GDC). For each sample, we identified if a patient harbored at least one nonsense, frameshift insertion, or frameshift deletion mutation in APC which were already annotated per GDC.

## De novo* motif enrichment*

For all sets of 3′ UTRs, we downloaded available sequences [48]. We then utilized the R package memes version 5.5.0 [29], input the longest annotated 3′ UTR per gene, and ran MEME analysis with default settings for ungapped de novo motif identification and enrichment analyses.

### Generation of an empirical distribution of expected rates of 3’ UTR lengthening

Genes identified as direct APC binders were downloaded from supplementary data published from previous mouse neurogenesis HITS-CLIP data [26]. Of the identified genes, we identified the 210 human orthologs present in our colorectal adenocarcinoma APA analyses. We then assess the fraction of those genes that displayed significant shortening, lengthening, or no change in APC loss-of-function versus no loss-of-function mutations. We then randomly subsampled 10,000 random groupings of 210 genes from the differential APA analysis to generate an empirical distribution of the expected fraction of genes that would be expected to be lengthened, unchanged, or shortened by random chance.

### HITS-CLIP data analysis

Previously published HITS-CLIP data was downloaded from NCBI SRA (SRP042131) [26]. Data was then processed and analyzed in accordance with the DEWseq data preprocessing designed for eCLIP data [49]. In brief, FASTQ files were mapped and aligned to an annotated mouse genome (mm10) using the default setting in HISAT2 [50]. Crosslink sites were then extracted using HTSeq-CLIP using default settings with a sliding window approach with default window size and step settings [51]. Count matrices were then loaded into R and the reads per sliding scale window were pooled across all 4 available replicates to maximize statistical power. We then restricted analyses to sites containing at least 50 mapped reads. We then obtained the sequences of 500 bp up and downstream of the sliding window utilizing Biostrings [52] and then screened the up and downstream sequences for the first instance of a canonical poly(A) site relative to the HITS-CLIP binding site. For each HITS-CLIP target, we identified the nearest up and downstream canonical poly(A) signal sequences, AATAAA or ATTAAA [3, 31].

Annotated poly(A) sites were downloaded from a publicly available resource, PolyADB v3 [32]. We measured the distance from annotated APC binding sites to each annotated poly(A) site in a given 3′ UTR. Poly(A) signal sequences were then binned as either proximal (most 5′ within a given 3′ UTR), distal (most 3′ within a given 3′ UTR), and intermediate (all other poly(A) signals within the 3′ UTR).

### EGFR pathway expression and summary scoring

The canonical EGFR signaling pathway genes were identified from the Gene Set Enrichment Analysis (GSEA) database [53, 54]. For each sample, we assessed gene expression as described above (transcripts per million). For each individual sample, we also assessed the overall level of APC expression (transcripts per million) per sample and divided the TCGA low-grade glioma cohort into quartiles based on APC expression. We then calculated the individual Z score per gene within the EGFR signaling pathway. We then summed the *Z* scores of all canonical EGFR genes as a summary score of average EGFR signaling for each sample, where a higher score reflects on average higher expression of canonical EGFR genes and a lower score reflects on average lower expression of canonical EGFR genes.

## Supplementary Information

Additional file 1: Figures S1-S5.

Additional file 2: Table S1. Pan-cancer genome wide Pearson correlation of gene expression and median 3′ UTR. Individual Pearson correlation coefficients (*R*) and *P* values associated between gene expression and sample median 3′ UTR length. For each gene the ensemble gene ID and gene name are listed, along with the R and p value per TCGA cancer cohort identified with the standard TCGA subtype acronyms.

Additional file 3: Table S2. Summary Pearson c orrelation between gene expression and median 3′ UTR length. Summary data generated for Additional file 1: Figure S1D reflecting the mean and standard deviation of Pearson correlation coefficients generated for all 29 TCGA cancer cohorts analyzed.

Additional file 4: Table S3. Differentially polyadenylated transcripts in colorectal andeocarcinoma samples harboring *APC* nonsense or frameshift mutations. Quantification of the average 3′ UTR length per geen in samples with or without nonsense or frameshift mutations in APC. For each gene, the median 3′ UTR length per group is identified which was quantified using DaPars [11].

Additional file 5: Table S4. Differentially polyadenylated transcripts in human colon orangoids with *APC* gene knockout. APAlyzer analysis output [28] indicating the difference in the log_2_(distal/proximal reads) between human colorn organoids with or without APC knockout.

Additional file 6: Table S5. APC HITS-CLIP renanalysis target enrichment. Reanalaysis of APC HITS-CLIP data from Preitner et al. 2014 [26]. Enriched RNA targets with specific window, number of reads quantified per replicate, and specific gene annotation if enriched within a genic region. Data shown for all targets with more than 50 reads per target window total across all four replicates.

Additional file 7: Table S6. Differentially polyadenylated transcripts in low grade glioma samples with high or low *APC* gene expression. Quantification of the average 3′ UTR length per geen in samples with either high or low APC gene expression. For each gene, the median 3′ UTR length per group is identified which was quantified using DaPars [11].

Additional file 8: Review history.

## Data Availability

RNA-seq data generated by TCGA were downloaded from the Genomic Data Commons (GDC) and the Cancer Genomics Hub (CGHub). Human colon organoid RNA-seq data generated by Ringel et al., 2020 were downloaded from the Gene Expression Omnibus (GSE145185) [30, 55]. HITS-CLIP data from Preitner et al., 2014 were downloaded from the Sequence Read Archive (SRP042131) [26, 56]. Data from all analyses, including gene expression and 3′ UTR level measurements and correlations generated for this paper, are included in the supplementary materials as follows. Pearson correlation coefficients from gene expression and median 3′ UTR per dataset and the associated *P* value are included in Additional file 2: Table S1. Mean Pearson correlation coefficients (*R*) across all analyzed datasets are included in Additional file 3: Table S2. Median 3′ UTR length per colorectal adenocarcinoma generated using the DaPars algorithim are included in Additional file 4: Table S3, and the imputed values for that same dataset but using APAlyzer are included in Additional file 5: Table S4. Reanalysis of HITS-CLIP data from Prietner et al. 2014 [26] are included in Additional file 6: Table S5. Differential APA analysis using DaPars of TCGA LGG samples with high or low APC expression are in Additional file 7:Table S6. No other scripts or software were utilized other than those mentioned in the Methods section.
