# Supplementary material for: APC mutations dysregulate alternative polyadenylation in cancer
Source: Genome Biol. 2024 Oct 7;25:255. doi: 10.1186/s13059-024-03406-4 (PMC11457450; doi:10.1186/s13059-024-03406-4)
Supplement: Supplementary file 1 — Additional file 1: Figures S1-S5. [file 13059_2024_3406_MOESM1_ESM.pdf]

FIGURE S1

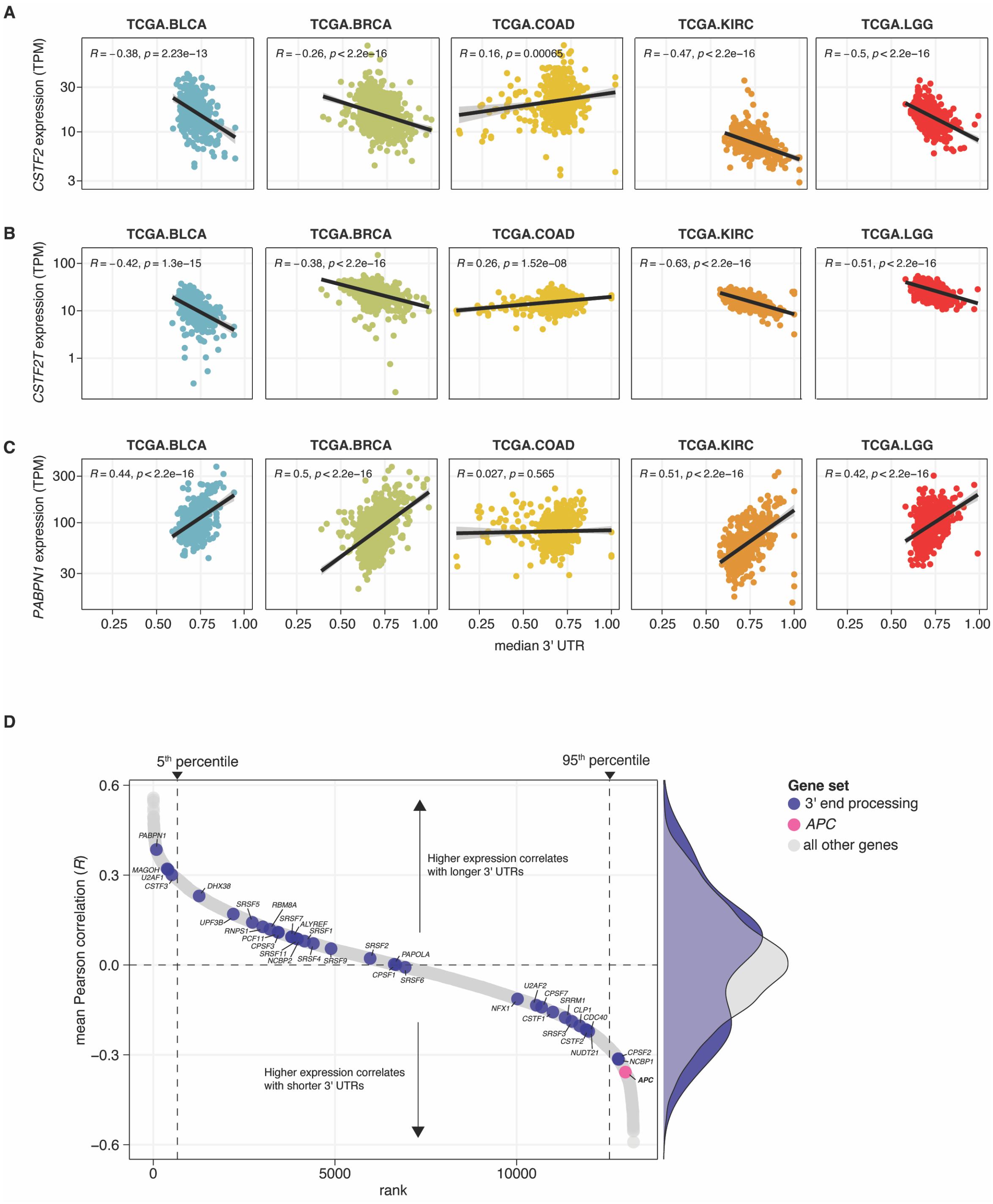

Figure S1. Canonical poly(A) site selection regulators correlate with global 3′ UTR length in all cancer subtypes except colorectal adenocarcinoma.

(**A-C**) Scatterplots of median 3′ UTR length per sample versus gene expression of the indicated gene (TPM). Plots shown for known poly(A) site regulators *CSTF2*, *CSTF2T*, and *PABPN1*. *R* and *p* values are calculated from Pearson correlation.

(**D**) Waterfall plot of mean Pearson correlation coefficient (*R*) of gene expression and median 3′ UTR length per coding gene across 30 TCGA datasets. Genes are colored as blue if previously identified as involved in 3′ end processing or gray if not, and pink indicates *APC*. Dotted lines indicate 5th and 95th percentiles of all gene level Pearson correlations.

FIGURE S2

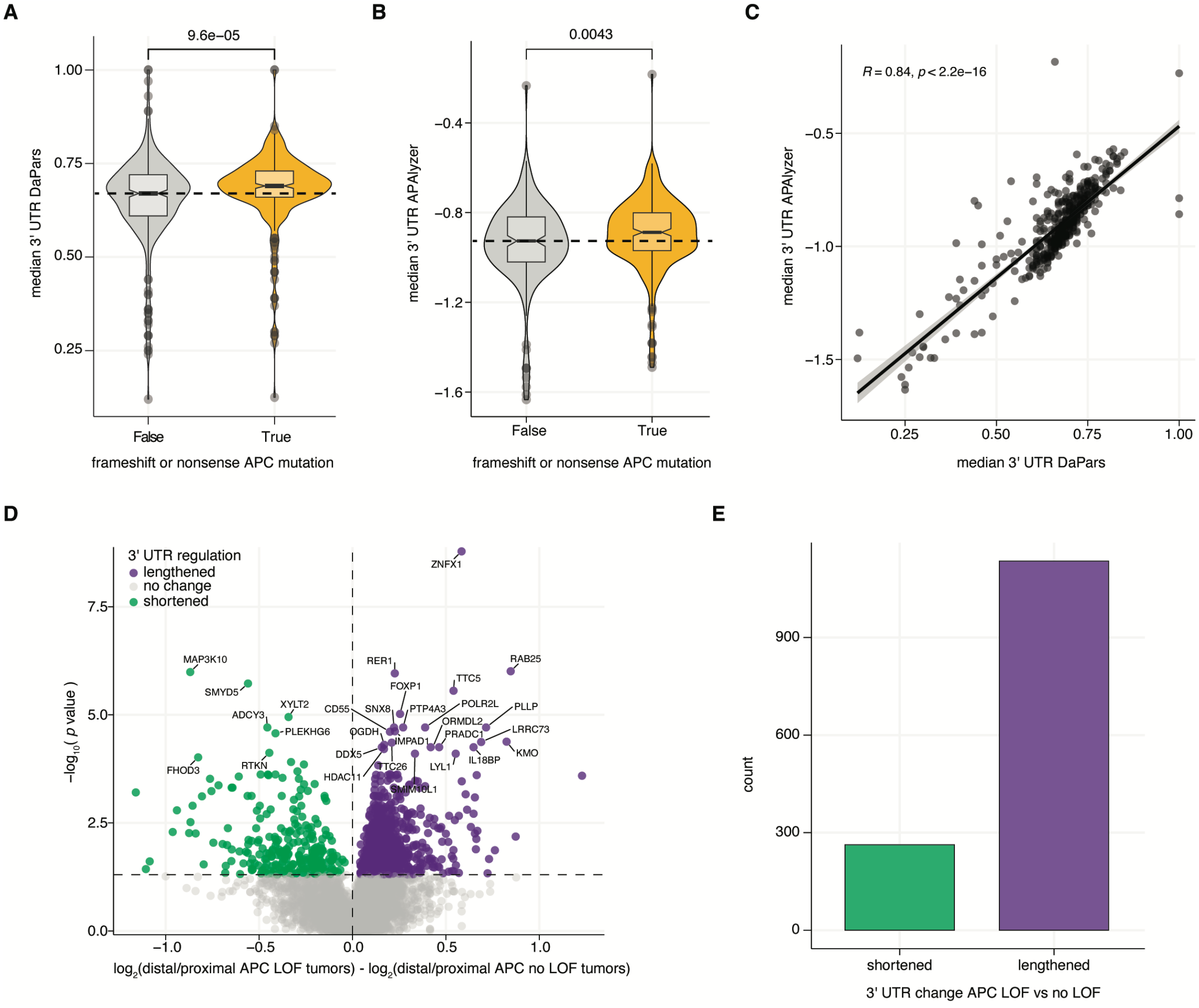

Figure S2. APC loss-of-function mutations are associated with global 3′ UTR lengthening.

(**A**) Violin plots of median 3′ UTR per sample (calculated using DaPars pipeline) with a detected APC nonsense or frameshift mutation (yellow) compared to samples with either missense, silent or wild-type APC (gray) in the TCGA colorectal adenocarcinoma dataset. *P* value from two-sided Wilcoxon rank-sum test.
(**B**) Violin plots of median 3′ UTR per sample (calculated using APAylzer pipeline) with a detected APC nonsense or frameshift mutation (yellow) compared to samples with either missense, silent or wild-type APC (gray) in the TCGA colorectal adenocarcinoma dataset. *P* value from two-sided Wilcoxon rank-sum test.
(**C**) Scatter plot of median 3′ UTR value per colorectal adenocarcinoma sample calculated using the DaPars or APAlyzer algorithm. *R* and *p* values are calculated from Pearson correlation.
(**D**) Volcano plot comparing 3′ UTR length in APC nonsense or frameshift samples versus samples without a detected APC nonsense or frameshift mutation in the TCGA colorectal adenocarcinoma dataset. 3′ UTR measurements are calculated using the APAlyzer algorithm.

Individual UTRs are indicated as shortened (green), lengthened (purple) or no change (gray) based on a *p* value < 0.05 using a two-sided Wilcoxon rank-sum test.
(**E**) Bar plot of significantly altered lengthening (purple, n = 1135) or shortening events (green, n = 263) in APC nonsense or frameshift colorectal adenocarcinoma samples.

FIGURE S3

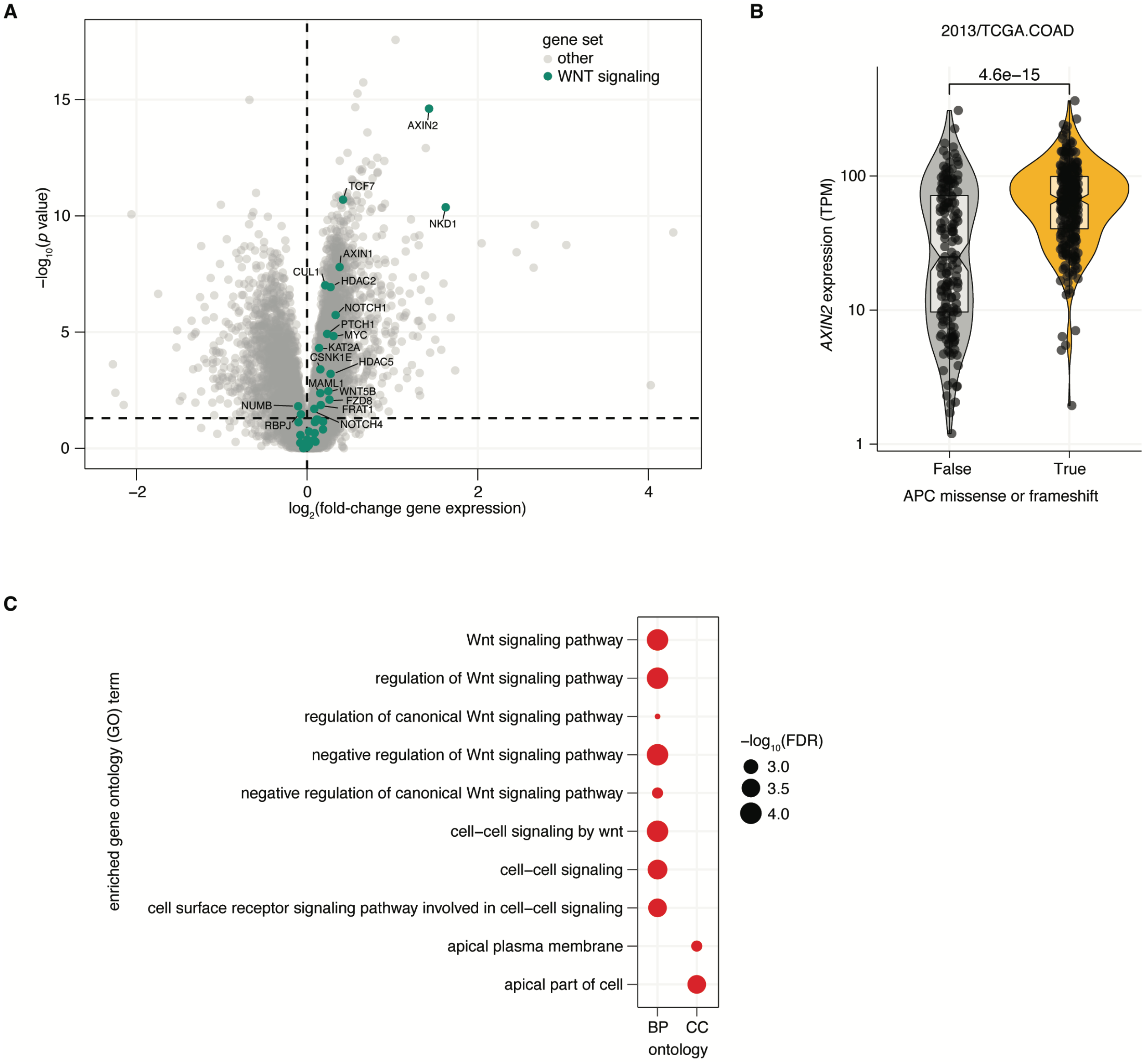

Supplemental Figure 3. Patient and human colon organoid differential gene expression analyses demonstrate expected activation of canonical WNT signaling.

(**A**) Volcano plot of the $\log_2$(fold-change gene expression) gene expression detected comparing colorectal adenocarcinoma samples from 2013/TCGA.COAD with or without and APC nonsense or frameshift mutation. WNT signaling genes are labeled in green.
(**B**) Violin plot of gene expression (transcripts per million, TPM) of colorectal samples lacking a nonsense or frameshift mutation in APC (gray) or colorectal samples with a nonsense or frameshift mutation in APC (yellow) of the canonical WNT signaling gene *AXIN2*. *P* value reflective of a two-sided Wilcoxon rank-sum test.
(**C**) Gene ontology (GO) analysis of genes with significantly increased expression in samples with a frameshift or nonsense mutation in APC from the 2013 TCGA colorectal adenocarcinoma dataset compared to colorectal samples lacking a nonsense or frameshift mutation in APC. Top 10 most significantly enriched GO terms are plotted, and the terms are plotted as being

biological processes (BP) or cellular compartments (CC). Points are scaled to the $-\log_{10}$(false discovery rate, FDR).

FIGURE S4

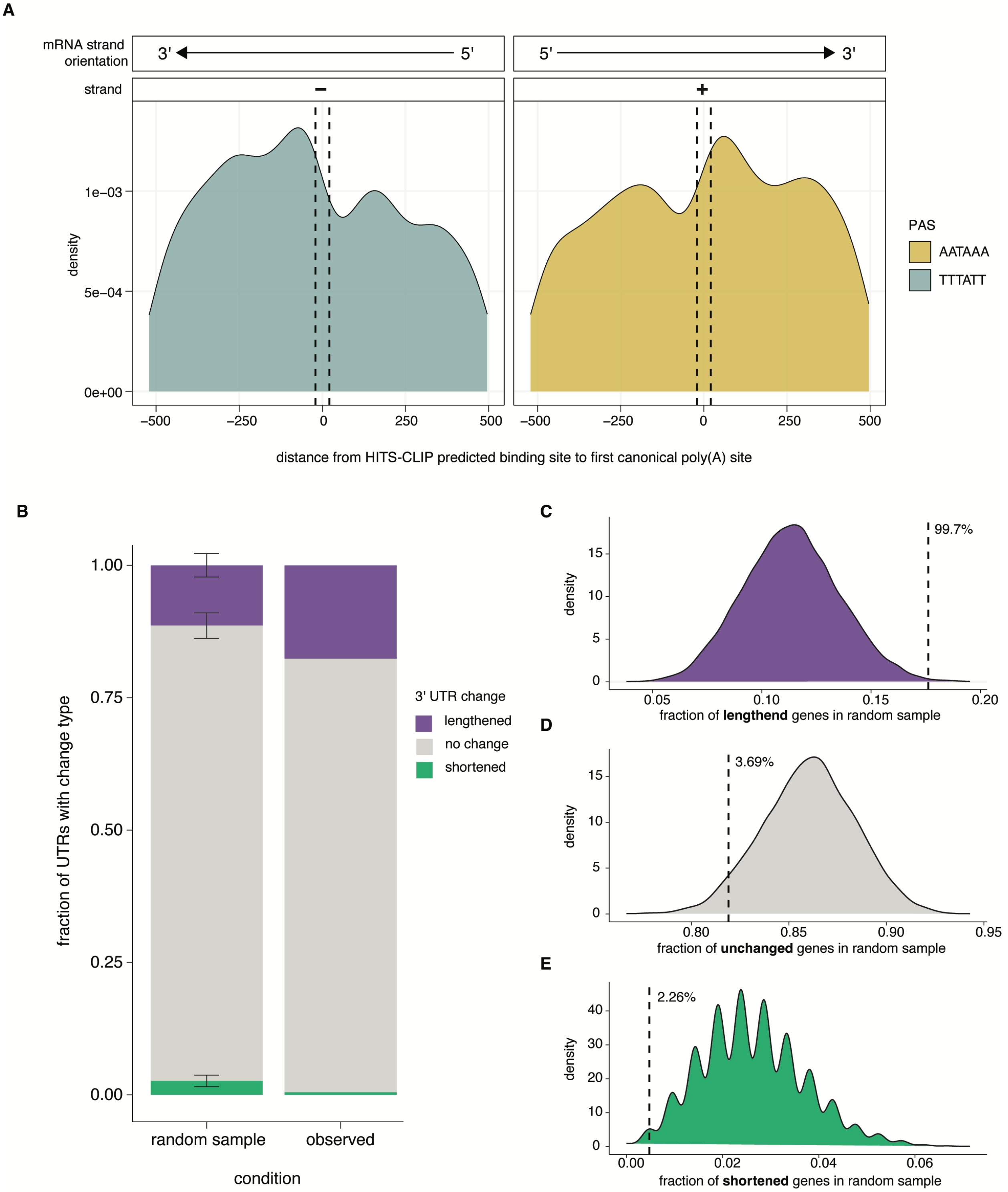

Figure S4. Reanalysis of mouse APC HITS-CLIP data and correlation with human data.

(**A**) Strand-specific density plots of aggregated data shown in **Figure 4B**. Enrichment of the canonical poly(A) signal sequence AATAAA (TTTATT on minus strand, reverse compliment of

canonical sequence). Plots colored by exact orientation of the canonical poly(A) signal sequence (PAS) assessed based on strandedness (yellow for AATAAA, blue for TTTATT). mRNA strand orientation indicates 5’ to 3’ orientation of analyzed genes for that strand. Dotted lines indicate 50 bp window of identified HITS-CLIP binding.
(**B**) Stacked bar plot of the fraction of observed significance calls, meaning identified as significantly lengthened, significantly shortened, or unchanged in APC loss-of-function colorectal adenocarcinoma samples, for the 210 genes identified as APC targets from Preitner et al (observed) versus an empirical distribution generated from subsampling randomly selected groups of genes from all genes analyzed in our differential APA analysis (random sample).
(**C-E**) Density plots of the empirical distributions generated for the fraction of 210 randomly sampled genes from our differential APA analysis to be lengthened (purple), unchanged (gray), or shortened (green). These were constructed by randomly sampling the output of all genes analyzed for differential APA anlysis (supplemental figure 3) 10,000 times and computing the fraction of genes with each type of change. Vertical dotted line indicates the observed fraction of genes with the indicated significance call, and the value represents the percentage of all randomly sampled data with a small fraction than the observed value from the Preitner et al gene list.

FIGURE S5

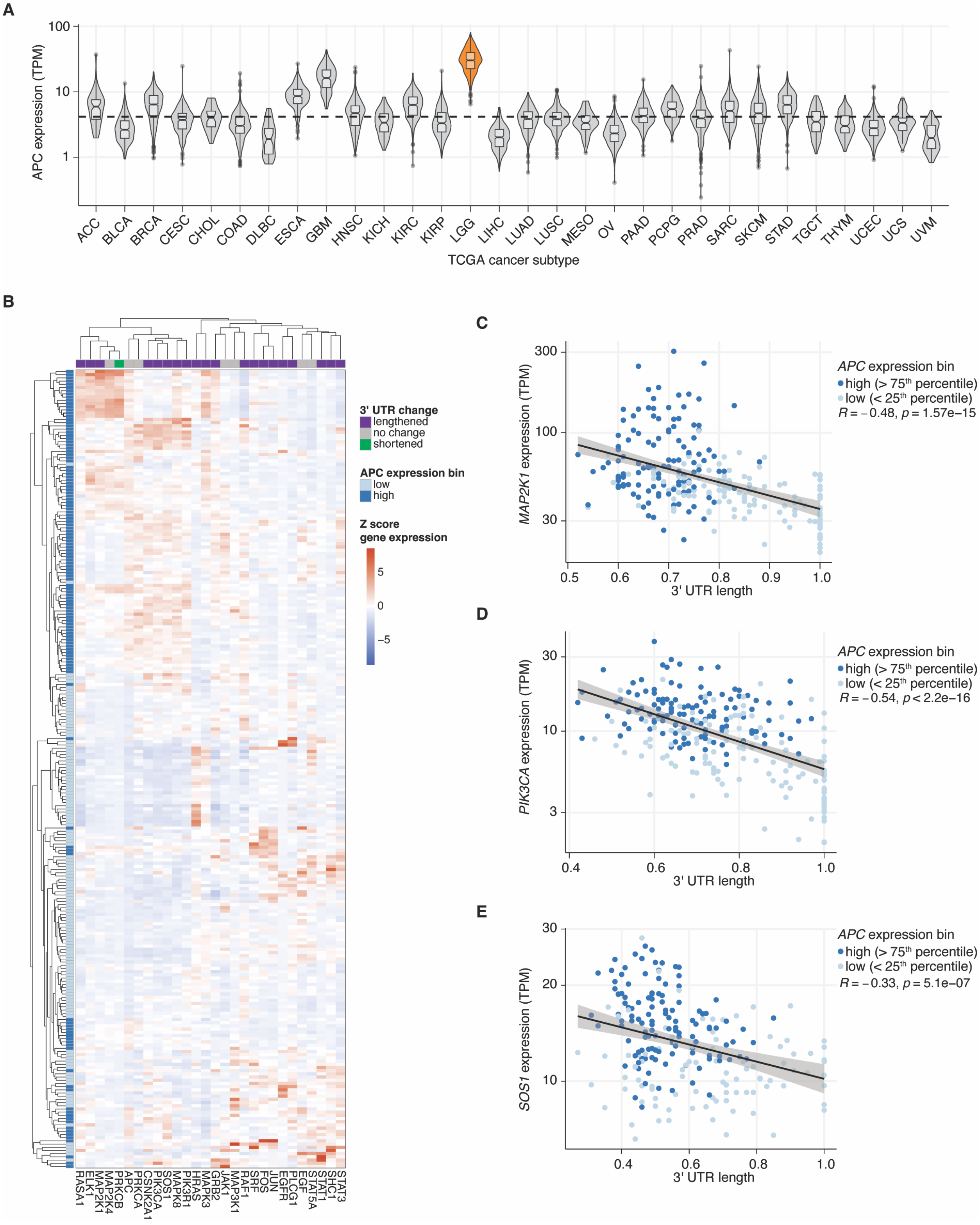
A
APC expression (TPM)
100
10
1
ACC BLCA BRCA CESC CHOL COAD DLBC ESCA GBM HNSC KICH KIRC KIRP LGG LIHC LUAD LUSC MESO OV PAAD PCPG PRAD SARC SKCM STAD TGCT THYM UCEC UCS UVM
TCGA cancer subtype
B
3' UTR change
lengthened
no change
shortened
APC expression bin
low
high
Z score
gene expression
5
0
−5
RASA1 ELK1 MAP2K1 MAP2K4 PRKCB APC PRKCA CSNK2A1 PIK3CA SOS1 MAPK8 PIK3R1 HRAS MAPK3 GRB2 JAK1 MAP3K1 RAF1 SRF FOS JUN EGFR PLCG1 EGF STAT5A STAT1 SHC1 STAT3
C
MAP2K1 expression (TPM)
300
100
30
0.5 0.6 0.7 0.8 0.9 1.0
3' UTR length
APC expression bin
high (> 75th percentile)
low (< 25th percentile)
R = −0.48, p = 1.57e−15
D
PIK3CA expression (TPM)
30
10
3
0.4 0.6 0.8 1.0
3' UTR length
APC expression bin
high (> 75th percentile)
low (< 25th percentile)
R = −0.54, p < 2.2e−16
E
SOS1 expression (TPM)
30
20
10
0.4 0.6 0.8 1.0
3' UTR length
APC expression bin
high (> 75th percentile)
low (< 25th percentile)
R = −0.33, p = 5.1e−07

Figure S5. APC expression correlates with APA of EGFR signaling genes in low grade glioma.

(**A**) Violin plot of APC expression by TCGA cancer subtype. Violin plot in orange indicates low grade glioma (LGG) expression data. Dashed line represents median APC gene expression across all assayed TCGA cohorts.
(**B**) Heatmap of gene expression of 28 EGFR signaling cascade genes. Samples are colored by APC expression bin as low (light blue <25th percentile) or high (dark blue >75th percentile). Change in 3′ UTR length per gene indicated (purple = lengthening, gray = no change and green = shortening in low grade glioma TCGA cohort comparing high versus low APC gene expression samples).
(**C-E**) Scatter plot of gene level 3′ UTR length versus gene expression of *MAP2K1*, *PIK3CA* and *SOS1* (all EGFR signaling genes) with samples colored by APC expression bin. *P* value and *R* from Pearson correlation.
