# Supplementary material for: APC mutations dysregulate alternative polyadenylation in cancer
Source: Genome Biol. 2024 Oct 7;25:255. doi: 10.1186/s13059-024-03406-4 (PMC11457450; doi:10.1186/s13059-024-03406-4)
Supplement: Supplementary file 8 — Additional file 8: Review history. [file 13059_2024_3406_MOESM8_ESM.docx]

Review History

**First round of review**

**Reviewer 1**

**Were you able to assess all statistics in the manuscript, including the appropriateness of statistical tests used?**

Yes, and I have assessed the statistics in my report.

**Comments to author:**

The authors of this manuscript investigated the role of APC mutations in alternative polyadenylation. They performed an initial screen in which they compared usage of proximal versus distal polyadenylation sites with the expression of individual genes among tumors. A clear correlation was observed for genes previously known to affect choice of polyadenylation site. These relationships were similar among tumor types, with the exception of colorectal cancer, which was an outlier. They discovered that colorectal adenocarcinomas with mutations in APC had global 3' UTR lengthening compared with colorectal adenocarcinomas predicted to have APC function. Analysis of human colon organoids revealed that APC knockout was associated with lengthening of 3' UTRs when a motif predicted to be an APC binding site was present. In glioblastoma, APC expression was associated with changes in 3' UTR length and the expression of genes in key signaling pathways. APC is a critically important tumor suppressor and the results shed new light on its function.

Comments

1. The introduction is very short. The authors may want to cite some key papers that show a connection between APA, proteins that regulate APA, and cancer incidence or progression to better establish the state of knowledge of the field.

2. The authors only use Da Pars to impute 3' UTR length in Fig 1. The authors use 2 different methods in Fig 2 and the data look similar. They might want to consider using APAlyzer for the Fig 1 data as well.

3. In the results section Fig 1B, the authors perform Pearson correlations within each cancer subtype. It would be helpful to clarify that for the reader in the text.

4. In Fig 1C, it would be helpful for the authors to clarify that each point in the figures represents a tumor.

5. It is unfortunate that the organoid data do not reveal a lengthening with APC knockdown in all of the transcripts without the focus on the transcripts with the motif because the motif is not well-established (no actual pull-down data) and the number of motifs to be included seems arbitrary.

6. Given that the analysis of APC data in the organoids does not reveal a significant change overall in 3' UTR use in epithelial cells with APC knockdown, what do the authors think explains the separation of colorectal cancer from the other tumor types in Fig 1E? Is it possible that it reflects cells other than the tumor cells?

7. It's surprising that in the low-grade glioma, APC knockdown does affect 3' UTR use, but not in the colon organoids. How do the authors interpret this, especially given that the colon adenocarcinomas look so different from the other tumors in Fig 1E.
8. In the gene expression values in APC high and APC low glioblastomas in Fig 4F, it seems low APC expression as would be expected for APC mutations, results in lower levels of these signaling pathway genes, which seems like the opposite of expectations if APC inactivation is part of the tumorigenesis process.

9. It is very difficult to determine whether APC's role in cancer reflects its effects on the Wnt pathway or APA. Can the authors please speak to this question of whether there is any biological effects of these changes in APA site selection with APC mutation?

10. The discussion is also very short. The authors could comment on the topics highlighted in this review such as the direction of the effect on the cell signaling genes, the differences between colon cancer and glioma, and how it might be possible to distinguish the effects of Wnt signaling versus APA on tumor incidence and progression.

**Reviewer 2**

**Were you able to assess all statistics in the manuscript, including the appropriateness of statistical tests used?**

No, I do not feel adequately qualified to assess the statistics.

**Comments to author:**

In this manuscript, Gable et al analyzed TCGA and other published datasets and found that colorectal cancer display unique APA and regulator expression relationship and identified a potential role for APC in regulating alternative polyadenylation (APA). These finding, if substantiated, are quite interesting and important. However, in my opinion, their conclusion is not fully supported by the data. Here are my comments:

1. Loss of function in APC seems to cause predominantly 3' UTR lengthening (Fig. 2A). However, APC knockout caused roughly equal number of 3' UTR lengthening and shortening events (Fig. 4A). How to reconcile these differences?
2. The authors argue that APC directly regulates APA by binding to 3' UTR sequences. Since the APA CLIP data is available, more detailed analysis should be done to determine the enrichment and position of APC-binding sites relative to the regulated APA sites.
3. The APA changes described, including Fig. 3C,E,G and Fig. 4D all seemed quite modest. If the authors could provide additional evidence for the functional importance of these APA changes (in addition to the ontology analysis), it would boost the significance of the results.

**Reviewer 3**

**Were you able to assess all statistics in the manuscript, including the appropriateness of statistical tests used?**

Yes, and I have assessed the statistics in my report.

**Comments to author:**

The manuscript is generally well-written. I only have a few comments:

Were the analyzed samples checked for RNA integrity? This can dramatically skew the gene expression and 3'UTR length measurements.

Were the 3'UTRs included in the gene expression estimation? Shortening and lengthening could also skew gene expression measurements.

When checking the de novo motif enrichment of lengthened 3′ UTRs the authors find that the top 8 motifs are largely G and C rich. They claim that this is concordant with GUGU they expected, but that is not the case. There is no need to misrepresent these results.

Fig. 1E - esophageal is misspelled

IGV screen shots are hard to evaluate when filled curves of different colors are not transparent.

The positive controls presented are not the most broadly known regulators of 3'UTR length (CSTF2/T for example). What about CFIm?

Furthermore even for the positive controls, all patterns of correlation with 3'UTR ;ength changes (positive/negative/no
correlation) are observed, depending on the cancer. Is there evidence that these patterns are so variable or do they represent study-specific biases?

It appears that the authors found gene expression to be positively correlated with median 3'UTR, which is opposite to the general expectation. Can the authors explain this discrepancy?

**Authors Response**

**Point-by-point responses to the reviewers’ comments:**

**Reviewer #1:**

The authors of this manuscript investigated the role of APC mutations in alternative polyadenylation. They performed an initial screen in which they compared usage of proximal versus distal polyadenylation sites with the expression of individual genes among tumors. A clear correlation was observed for genes previously known to affect choice of polyadenylation site. These relationships were similar among tumor types, with the exception of colorectal cancer, which was an outlier. They discovered that colorectal adenocarcinomas with mutations in APC had global 3' UTR lengthening compared with colorectal adenocarcinomas predicted to have APC function. Analysis of human colon organoids revealed that APC knockout was associated with lengthening of 3' UTRs when a motif predicted to be an APC binding site was present. In glioblastoma, APC expression was associated with changes in 3' UTR length and the expression of genes in key signaling pathways. APC is a critically important tumor suppressor and the results shed new light on its function.

*Reply: We thank the reviewer for their thoughtful comments and review of our paper.*

Comments

1. The introduction is very short. The authors may want to cite some key papers that show a connection between APA, proteins that regulate APA, and cancer incidence or progression to better establish the state of knowledge of the field.

*Reply: We thank the reviewer for this comment and have added additional background to the introduction in the revised manuscript.*

2. The authors only use Da Pars to impute 3' UTR length in Fig 1. The authors use 2 different methods in Fig 2 and the data look similar. They might want to consider using APAlyzer for the Fig 1 data as well.

*Reply: We agree that it is important to confirm that computational methods used for inference, namely the DaPars and APAlyzer methods, yield concordant results. We previously validated the concordance between inference of 3' UTR length by DaPars and APAlyzer based on RNA-seq data in our recent manuscript reporting that dysregulated APA contributes to tumor growth in melanoma (Gabel et al, Nature Communications, 2024). Those studies confirmed that DaPars and APAlyzer yield highly* *concordant estimates of 3 UTR length across libraries from distinct RNA isolation and preparation methods. For example, the below scatter plot (from our previous study) demonstrates the concordance of 3 UTR length inference across the TCGA cutaneous melanoma cohort:*

***Concordance of median 3’ UTR length analyses with distinct computational pipelines.*** *Each dot represents a TCGA cutaneous melanoma RNA-seq sample. Global median 3’ UTR was imputed using either DaPars or APAlyzer and samples were then binned into terciles (short, medium, or long UTRs). Green indicates the sample was classified in the same bin by either algorithm.*

*Reply: These studies from our previous manuscript establish that DaPars and APAlyzer yield highly concordant 3' UTR length inferences and are both excellent choices for algorithms when studying APA based on RNA-seq data.*

3. In the results section Fig 1B, the authors perform Pearson correlations within each cancer subtype. It would be helpful to clarify that for the reader in the text.

*Reply: We thank the reviewer for this comment. We revised the text to clarify this point.*

4. In Fig 1C, it would be helpful for the authors to clarify that each point in the figures represents a tumor.

*Reply: We thank the reviewer for this comment. We revised the figure legend to clarify this point.*

5. It is unfortunate that the organoid data do not reveal a lengthening with APC knockdown in all of the transcripts without the focus on the transcripts with the motif because the motif is not wellestablished (no actual pull-down data) and the number of motifs to be included seems arbitrary.

*Reply: We agree with the reviewer’s point that the association between APC knockdown and 3' UTR lengthening is clearly evident for transcripts containing enriched motifs identified via motif enrichment analyses in primary human cancer samples but not when this restriction is removed. One possible explanation for why the trend is so readily evident in cancer samples but not in the organoid data is that the RNA-seq data in these organoid experiments was generated only 24 hours following induction of knockdown, a relatively short time scale for reduced APC levels to affect 3′ UTR usage and then have that altered 3′ UTR usage become apparent after sufficient mRNA turnover occurs. We speculate that at longer time scales, the lengthening phenotype might be more readily apparent without restricting to transcripts containing the enriched motifs that we identified. While this hypothesis cannot be tested without additional experimental evidence, we are encouraged that the data hint at sequence-specific lengthening* *even within 24 hours of knockdown. We edited the Discussion to note this important caveat/limitation to our study:*

*There are several limitations to our work that are important to note. First, the association between APC knockdown and 3′ UTR lengthening in organoids is clearly evident for transcripts containing enriched motifs identified via motif enrichment analyses in primary human cancer samples but not when this restriction is removed. We hypothesize that this may be due to the relatively short interval (24 hours) between APC knockdown and collection for RNA-seq but cannot directly test this hypothesis without additional experimental evidence.*

*In the revised manuscript, we include additional new data providing orthogonal evidence supportive of a direct role for APC in affecting APA. Specifically, in response to a suggestion from reviewer #2, we reanalyzed the APC HITS-CLIP data presented in Preitner et al 2014 to assess if there was a relationship between APC binding and distance to regulated poly(A) sites. We first downloaded and reanalyzed the HITS-CLIP data generated by Preitner et al using*

*HTSeq-CLIP (Sahadevan et al., 2023). We then assessed the distance from enriched APC RNA-binding sites to canonical poly(A) signal sequences (AATAAA, ATTAAA) to see if there was an obviously detectable relationship between APC binding site and proximity to poly(A) cleavage sites. We found a striking enrichment in poly(A) sites adjacent to APC binding sites (****Figure 4A-B****; below).*

***APC RNA-binding sites are frequently proximal to canonical poly(A) signal sequences****. Left, summary cartoon of analysis pipeline to quantify distance between APC binding and nearest poly(A) signal sequence. Right, density plot of the upstream and downstream nearest poly(A) site relative to each enriched APC RNA-binding site. Dotted lines indicate window of enriched APC binding. There is an maxima of canonical poly(A) signal sequences centered at +62.19 nucleotides downstream of enriched APC binding sites.*

*We then assayed if orthologous genes identified as APC binding targets in Preitner et al displayed more drastic lengthening phenotypes in our cancer transcriptome analysis. We saw that when APC binding targets displayed significantly more dramatic lengthening phenotypes than genes not identified as binding targets (****Figure 4C****; below).*

***UTRs bound to APC display significantly more dramatic lengthening phenotypes****. Boxplots of differential 3’ UTR length in colorectal adenocarcinoma samples (COAD) comparing samples with or without an APC loss-of-function mutation of genes identified as APC binding targets (blue) or not binding targets (white). In this plot, a more positive value indicates a longer 3’ UTR in APC loss-of-function samples, and a more negative value indicates a shorter 3’ UTR. P value from a two-sided Wilcoxon Rank-Sum test.*

*We next wanted to assess if the identified RNA binding targets of APC were more likely to be significantly lengthened than we would expect by random chance. There were 210 identified genes in Preitner et al with a human ortholog and a quantified difference in 3’ UTR length in the TCGA COAD dataset we analyzed (Supp. Table 3). Of those, 37 (17.6%) displayed lengthening,*

*172 (81.9%) displayed no significant change, and 1 (0.4%) displayed significant shortening (****Supp. Figure 4B****; below). We then subsampled the differential APA analysis 10,000 times to build an empirical distribution of what percentages of lengthened, unchanged, or shortened 3’ UTRs we would expect based on random chance. We found that the observed data, where 17.6% of the genes displayed significant lengthening, was a higher fraction than 99.7% of all randomly sampled data (****Supp. Figure 4C****). This is highly suggestive that the 3’ UTRs identified in Preitner et al display significantly more 3’ UTR lengthening than we would expect by random chance. This is especially notable considering this data was originally from mouse neurogenesis data. Thus, it is tempting to speculate a degree of conservation in the binding site and binding targets of APC, which is also supported by the concordance of C- and G- rich motifs identified by Preitner et al HITS-CLIP data as well as our de novo motif enrichment analyses (****Figure 2C****).*

***Generation of an empiric distribution of expected alterations in 3’ UTR length by random*** ***chance****. Stacked bar blot representing the expected distribution of lengthened, unchanged or shortened 3’ UTRs by random chance () compared to the observed distribution from our dataset. Density plots of the empiric distributions for the lengthened (purple), unchanged (grey), or shortened (green) where X is the fraction of genes in a given random sample classified as such.*

*Dotted lines in each density plot represent the observed data from Preitner et al, and the*

*number represents the percent of simulated data with a smaller value than the observed data.*

*Overall, these new data provide strong evidence supporting the model that we propose.*

6. Given that the analysis of APC data in the organoids does not reveal a significant change overall in 3' UTR use in epithelial cells with APC knockdown, what do the authors think explains the separation of colorectal cancer from the other tumor types in Fig 1E? Is it possible that it reflects cells other than the tumor cells?

*Reply: We thank the reviewer for this insightful comment. We believe that the signal for differences in 3' UTR usage in colorectal adenocarcinoma samples are due to loss-of-function mutations in APC in tumor cells. This hypothesis is consistent with our analyses of the organoid data but agree with the reviewer that it would have been more satisfying to observe a strong lengthening phenotype without the restriction to* *transcripts containing enriched motifs. We hypothesize that a longer time scale for the APC knockdown experiment might have revealed just such a*

*stronger trend. However, as this is a hypothesis that we cannot test without performing experiments that are outside the scope of this computational study, we carefully note this limitation in the revised Discussion:*

*There are several limitations to our work that are important to note. First, the association between APC knockdown and 3' UTR lengthening in organoids is clearly evident for transcripts containing enriched motifs identified via motif enrichment analyses in primary human cancer samples but not when this restriction is removed. We hypothesize that this may be due to the relatively short interval (24 hours) between APC knockdown and collection for RNA-seq but cannot directly test this hypothesis without additional experimental evidence.*

7. It's surprising that in the low-grade glioma, APC knockdown does affect 3' UTR use, but not in the colon organoids. How do the authors interpret this, especially given that the colon adenocarcinomas look so different from the other tumors in Fig 1E.

*Reply: We thank the reviewer for this comment. We see a strong negative correlation between APC expression and median 3’ UTR length across all TCGA datasets (not just low-grade glioma) except for colorectal adenocarcinoma (see figure below and Supp. Figure 1D). We believe this arises from very frequent APC mutations in colorectal adenocarcinoma, leading to a pattern of poly(A) site selection that is distinct from other TCGA cancer subtypes.*

***APC expression is negatively correlated with global median 3' UTR****. Scatter plots of APC gene expression (TPM) versus median 3' UTR length. Each dot here represents an individual RNA-seq tumor sample from the respective cohort. Colorectal adenocarcinoma is a clear outlier. It additionally displays a significant positive correlation which, while interesting, is likely difficult to disentangle as APC expression levels and mutations interact in a variety of complex ways (Zhang & Shay, J. Natl Cancer Inst 2017).*

*Regarding the comparison between our analyses of the low-grade glioma cohort (and other cancer types) and the organoid data, we hypothesize that the association between APC expression and altered 3' UTR usage is more readily apparent in cancer sample than in the organoid data arises from the short time frame of the organoid experiment. The organoid RNAseq data was generated only 24 hours following induction of knockdown, a relatively short time scale for reduced APC levels to affect 3' UTR usage and then have that altered 3' UTR usage become apparent after sufficient mRNA turnover occurs.*

8. In the gene expression values in APC high and APC low glioblastomas in Fig 4F, it seems low APC expression as would be expected for APC mutations, results in lower levels of these signaling pathway genes, which seems like the opposite of expectations if APC inactivation is part of the tumorigenesis process.

*Reply: We thank the reviewer for this comment. In assessing levels of APC expression, we do not intend to assess the tumorigenicity of high or low APC expression status (and apologize if that wasn’t clear). Our primary goal for this analysis was to test whether low versus high APC expression was associated with 3’ UTR alterations that correlate with gene expression of known pathogenic genes in low grade glioma. While we find these data thought provoking (e.g., that expression levels of APC may alter poly(A) site selection and thus gene expression, which could potentially—to speculate—alter levels of genes implicated in pro-tumorigenic signaling), the analysis is exploratory. We have more clearly clarified the limitations of the data analysis in the revised Discussion:*

*Third, although we present exploratory analyses indicating that APC expression is correlated with alterations in APA as well as with expression of genes in pathways critical for tumor growth and survival, additional work is needed to assess the potential functional relevance of those changes to tumorigenesis.*

9. It is very difficult to determine whether APC's role in cancer reflects its effects on the Wnt pathway or APA. Can the authors please speak to this question of whether there is any biological effects of these changes in APA site selection with APC mutation?

*Reply: We thank the review for this insightful comment. We agree that given the many means by which APC affects molecular and cellular processes within tumors, it is difficult to know which might be most important in any given context. Given the well-described role for Wnt signaling in many tumor contexts, it seems reasonable to believe that the connection between APC and Wnt signaling is important. We also speculate that the link between APC and 3' UTR length that we describe here may also be relevant for tumorigenesis. However, given that our study is computational and primarily reliant upon correlative studies, we cannot make definitive statements about the functional relevance of the connections that we identify between APC and APA to tumorigenesis. We hope that our study will motivate other groups to pursue mechanistic studies to test some of the functional hypotheses arising from our study.*

10. The discussion is also very short. The authors could comment on the topics highlighted in this review such as the direction of the effect on the cell signaling genes, the differences between colon cancer and glioma, and how it might be possible to distinguish the effects of Wnt signaling versus APA on tumor incidence and progression.

*Reply: We thank the reviewer for this comment. We have added significant additional material to the discussion, with a goal of both describing and clarifying future directions as well as noting important limitations of the studies in this manuscript.*

**Reviewer #2:**

In this manuscript, Gable et al analyzed TCGA and other published datasets and found that colorectal cancer display unique APA and regulator expression relationship and identified a potential role for APC in regulating alternative polyadenylation (APA). These finding, if substantiated, are quite interesting and important. However, in my opinion, their conclusion is not fully supported by the data.

*Reply: We thank the reviewer for their positive comments about the potential importance of the findings presented in our manuscript. We agree that that there are several important limitations to our study, most arising from its status as a computational manuscript that is reliant upon correlative analyses of existing data, and have edited the Discussion to carefully note and describe the limitations of our study.*

*We also particularly thank the reviewer for their suggestion to analyze the CLIP data from Preitner et al, an excellent suggestion which substantially strengthened the revised manuscript.*

Here are my comments:

1. Loss of function in APC seems to cause predominantly 3' UTR lengthening (Fig. 2A). However, APC knockout caused roughly equal number of 3' UTR lengthening and shortening events (Fig. 4A). How to reconcile these differences?

*Reply: We agree with the reviewer’s point that the association between loss-of-function APC mutations is strongly associated with 3' UTR lengthening in primary human cancer samples but that this same association is only readily evident in the APC knockdown context in organoids when the analysis is focused on transcripts with enriched motifs. In particular, the association between APC knockdown and 3' UTR lengthening is clearly evident for transcripts containing enriched motifs identified via motif enrichment analyses in primary human cancer samples but not when this restriction is removed. One possible explanation for why the trend is so readily evident in cancer samples but not in the organoid data is that the RNA-seq data in these organoid experiments was generated only 24 hours following induction of knockdown, a relatively short time scale for reduced APC levels to affect 3' UTR usage and then have that altered 3' UTR usage become apparent after sufficient mRNA turnover occurs. We speculate that at longer time scales, the lengthening phenotype might be more readily apparent without restricting to transcripts containing the enriched motifs that we identified. While this hypothesis cannot be tested without additional experimental evidence, we are encouraged that the data hint at sequence-specific lengthening even within 24 hours of knockdown. We edited the Discussion to note this important caveat/limitation to our study:*

*There are several limitations to our work that are important to note. First, the association between APC knockdown and 3' UTR lengthening in organoids is clearly evident for transcripts containing enriched motifs identified via motif enrichment analyses in primary human cancer samples but not when this restriction is removed. We hypothesize that this may be due to the relatively short interval (24 hours) between APC knockdown and collection for RNA-seq but cannot directly test this hypothesis without additional experimental evidence.*

2. The authors argue that APC directly regulates APA by binding to 3' UTR sequences. Since the APA CLIP data is available, more detailed analysis should be done to determine the enrichment and position of APC-binding sites relative to the regulated APA sites.

*Reply: We thank the reviewer for this excellent suggestion. We reanalyzed the HITS-CLIP data presented in Preitner et al 2014 to assess, as suggested, if there was a relationship between APC binding and distance to regulated poly(A) sites. We first downloaded and reanalyzed the HITS-CLIP data generated by Preitner et al using HTSeq-CLIP (Sahadevan et al., 2023). We then assessed the distance from enriched APC RNA-binding sites to canonical poly(A) signal sequences (AATAAA, ATTAAA) to see if there was an obviously detectable relationship between APC binding site and proximity to poly(A) cleavage sites. We found a striking enrichment in poly(A) sites adjacent to APC binding sites (****Figure 4A-B****; below).*

***APC RNA-binding sites are frequently proximal to canonical poly(A) signal sequences****. Left, summary cartoon of analysis pipeline to quantify distance between APC binding and nearest poly(A) signal sequence. Right, density plot of the upstream and downstream nearest poly(A) site relative to each enriched APC RNA-binding site. Dotted lines indicate window of enriched APC binding. There is an maxima of canonical poly(A) signal sequences centered at +62.19 nucleotides downstream of enriched APC binding sites.*

*We additionally stratified HITS-CLIP binding sites by strand orientation (+/-) of the genes where the binding site is enriched in. We see that the canonical poly(A) site or the reverse complement of the canonical poly(A) site is significantly enriched downstream to the eCLIP site in the correct mRNA orientation based on the strandedness of the gene (****Supp. Figure 4A****; below).*

***APC RNA-binding sites are proximal to canonical poly(A) signal sequences****. Same data as above, split by which strand the coding gene is present on. We see that in both instances the canonical poly(A) signal sequence (AATAAA or the reverse compliment TTTATT) is significantly enriched on the 3’ side (downstream) of the identified APC binding site.*

*We then assayed if orthologous genes identified as APC binding targets in Preitner et al displayed more drastic lengthening phenotypes in our cancer transcriptome analysis. We saw that when APC binding targets displayed significantly more dramatic lengthening phenotypes than genes not identified as binding targets (****Figure 4C****; below).*

***UTRs bound to APC display significantly more dramatic lengthening phenotypes****. Boxplots of differential 3’ UTR length in colorectal adenocarcinoma samples (COAD) comparing samples with or without an APC loss-of-function mutation of genes identified as APC binding targets (blue) or not binding targets (white). In this plot, a more positive value indicates a longer 3’ UTR in APC loss-of-function samples, and a more negative value indicates a shorter 3’ UTR. P value from a two-sided Wilcoxon Rank-Sum test.*

*We next wanted to assess if the identified RNA binding targets of APC were more likely to be significantly lengthened than we would expect by random chance. There were 210 identified genes in Preitner et al with a human ortholog and a quantified difference in 3’ UTR length in the TCGA COAD dataset we analyzed (Supp. Table 3). Of those, 37 (17.6%) displayed lengthening, 172 (81.9%) displayed no significant change, and 1 (0.4%) displayed significant shortening (****Supp. Figure 4B****; below). We then subsampled the differential APA analysis 10,000 times to build an empirical distribution of what percentages of lengthened, unchanged, or shortened 3’ UTRs we would expect based on random chance. We found that the observed data, where 17.6% of the genes displayed significant lengthening, was a higher fraction than 99.7% of all randomly sampled data (****Supp. Figure 4C****). This is highly suggestive that the 3’ UTRs identified in Preitner et al display significantly more 3’ UTR lengthening than we would expect by random chance. This is especially notable considering this data was originally from mouse neurogenesis data. Thus, it is tempting to speculate a degree of conservation in the binding site and binding targets of APC, which is also supported by the concordance of C- and G- rich motifs identified by Preitner et al HITS-CLIP data as well as our de novo motif enrichment analyses (****Figure 2C****).*

***Generation of an empiric distribution of expected alterations in 3’ UTR length by random*** ***chance****. Stacked bar blot representing the expected distribution of lengthened, unchanged or shortened 3’ UTRs by random chance () compared to the observed distribution from our dataset. Density plots of the empiric distributions for the lengthened (purple), unchanged (grey), or shortened (green) where X is the fraction of genes in a given random sample classified as such.*

*Dotted lines in each density plot represent the observed data from Preitner et al, and the*

*number represents the percent of simulated data with a smaller value than the observed data.*

3. The APA changes described, including Fig. 3C,E,G and Fig. 4D all seemed quite modest. If the authors could provide additional evidence for the functional importance of these APA changes (in addition to the ontology analysis), it would boost the significance of the results.

*Reply: We agree with the reviewer that the APA alterations following APC knockdown in organoids are more modest than the striking changes that are associated with APC loss-of-function mutations in primary human cancers. We hypothesize that these more modest differences arise from the short time course used for the knockdown experiment (as described above in the response to comment #1). As 24 hours is a very short time frame for APC knockdown to have downstream consequences on 3' UTR length and then for those alterations to become evident as the cellular mRNA pool turns over, we believe that it is reasonable to expect more modest alterations in perceived 3' UTR usage in the organoid data than in the primary* *human cancer data, where APC mutations are acquired early during tumorigenesis (and so the sequenced mRNA pool reflects the consequences of long-term suppression of APC levels).*

*Regarding the potential functional importance of these changes, Figures 5D-G in the revised manuscript hint at the potential functional importance of the APA changes. The gene ontology analyses mentioned by the reviewer demonstrate that the APA changes are significantly enriched in genes involved in growth signaling pathways, including the EGFR signaling pathway, hinting at possible biological consequences. Fig. 5D-G in the revised manuscript demonstrate that those APA changes coincide with significant alterations in gene expression of specific key genes involved in enriched growth signaling pathways. This constitutes a very interesting correlative trend, whereby alterations in APC expression levels are strongly associated with altered expression of key components of signaling networks, potentially via fine-tuning of RNA levels via altered poly(A) site selection driven by APC. These changes are reflected in Figure 5D-G and Supp. Figure 5B-E (Figure 5E-G shown below).*

*(E) Violin plot of 3′ UTR length of several EGFR signaling genes in high versus low APC expressing stratified low grade glioma samples. P value from two-sided Wilcoxon rank-sum test. (F) Violin plot of gene expression of several EGFR signaling genes in high versus low APC expressing stratified low grade glioma samples. P value from two-sided Wilcoxon rank-sum test. (G) Violin plots of EGFR signaling pathway summary score (summed Z score of gene expression across 28 genes) per sample for low grade glioma samples stratified by APC expression bin. P value from two-sided Wilcoxon rank-sum test.*

**Reviewer #3:**

The manuscript is generally well-written.

*Reply: We thank the reviewer for the positive comments and helpful suggestions.*

I only have a few comments:

Were the analyzed samples checked for RNA integrity? This can dramatically skew the gene expression and 3'UTR length measurements.

***RNA-seq quality scores across samples****. RNA-seq quality scores assessed using RSeQC show no significant differences between APC loss of function and wild-type colorectal adenocarcinoma RNAseq samples.*

*Reply: We thank the reviewer for this comment and agree that RNA-seq quality can affect inference of 3' UTR length. It is therefore important to test whether differences in RNA-seq quality could potentially be a confounding factor influencing the results of our study. To address this, we analyzed RNA-seq quality by using RSeQC to assess RNA-seq coverage across gene bodies, a relevant proxy for RNA integrity given that these libraries were poly(A) selected (Wang et al, Bioinformatics 2012, PMID: 22743226). Using this analysis, samples with strong 3' end bias reflect lower quality RNA samples. As shown in the figure above, this analysis revealed that most samples show good coverage across the gene body (top-left panel). Importantly, we observed highly similar patterns of gene body coverage for the APC wild-type and loss-offunction samples used in our analyses (top-right panel), indicating that differences in RNA integrity do not confound the analyses in our manuscript.*

*We used gene-body coverage as a proxy for RNA integrity to conduct more fine-grained analyses as well. We binned samples based upon the 5' versus 3' end bias observed for each sample and compared median 3’ UTR lengths between APC loss of function or wild-type samples (see plot below). This analysis confirmed that APC loss of function mutations are associated with 3’ UTR lengthening even after controlling for RNA integrity.*

***APC loss-of-function mutations are associated with 3’ UTR lengthening irrespective of RNA integrity.*** *Samples from the TCGA COAD cohort were binned into terciles based on median RSeQC coverage scores across the gene body. Here bin 1 represents samples with relatively more 5’ end bias and bin 3 reflects samples with 3’ end bias. The association between APC mutations and altered 3' UTR length is strongest for samples in the middle tercile (which represent samples with the most even gene body coverage, consistent with high RNA integrity and an unbiased library preparation), as expected. Significance reflects a twosided Wilcoxon rank sum test.*

When checking the de novo motif enrichment of lengthened 3′ UTRs the authors find that the top 8 motifs are largely G and C rich. They claim that this is concordant with GUGU they expected, but that is not the case. There is no need to misrepresent these results.

*Reply: We thank the reviewer for this comment and apologize for the confusion. Our original manuscript contained a typo and did not fully describe the results reported in Preitner et al. Specifically, Preitner et al described three consensus motifs: one C-rich motif, one G-rich motif, and CUGU (not “GUGU”, which was a typo in our original manuscript). As the top motifs that we identified are G and C rich, these results are consistent with the consensus motifs reported by Preitner et al.*

Fig. 1E - esophageal is misspelled

*Reply: We thank the reviewer for catching this mistake and have fixed it accordingly.*

IGV screen shots are hard to evaluate when filled curves of different colors are not transparent.

*Reply: We thank the reviewer for pointing this out and have edited the figure accordingly.*

The positive controls presented are not the most broadly known regulators of 3'UTR length (CSTF2/T for example). What about CFIm?

*Reply: We thank the reviewer for this comment. CFIm is a multimeric protein complex including subunits of NUDT21, CPSF6, and CPSF7 (Rüegsegger et al Molecular Cell 1998). We have generated data for these correlations and included them as a summary plot in Supp. Figure 1D in the revised manuscript. This analysis shows that many of these genes also display correlations with median 3’ UTR length, though to varying degrees depending on the specific gene. In each case, colorectal adenocarcinoma displays the weakest or even a significant effect but in the opposite direction, again suggesting there is a unique feature of the COAD dataset distinguishing it from other TCGA cohorts (****Figure 1D-E****;* ***Supp. Figure 1A-D****).*

**Scatter plots of median 3’ UTR and additional 3’ end processing genes.**

Additional scatter plots of median 3’ UTR and gene expression for NUDT21, CPSF6, and CPSF7. We observe strong negative correlations with each of these genes, representing that in most cancer cohorts (COAD being the exception) higher expression of the respective gene is correlated with, on average, globally shortened 3’ UTRs.

*It is interesting to compare the Pearson correlation coefficients that we obtain for a selection of genes involved in 3’ end processing as well as APC across all TCGA cancer cohorts relative to correlation coefficients that we obtain for all other genes. For the negative control group (“other”, in grey), consisting of all genes not known to be involved in 3' end processing or APC, we observe a distribution centered at* *0, as expected. For the other illustrated genes, we observe significant departures from 0, consistent with functional roles in APA.*

**Pan-cancer Pearson correlation coefficients (*R*) of 3’ end processing genes.** For each illustrated gene, plot illustrates the Pearson correlation coefficients comparing median 3' UTR of 30 TCGA cancer subtypes compared to the distribution of all Pearson correlation coefficients generated across all cancer subtypes. *P* values are from a two-sided Wilcoxon rank sum test. Each dot represents a Pearson correlation coefficient from a TCGA cancer cohort.

*Finally, we note that as some genes’ exact relationship with global 3’ UTR length is still incompletely understood, the correlations that we computed could potentially suggest possible directions for future mechanistic studies. We therefore have included all correlations on a gene level and cancer subtype specific level in the supplementary data to allow others to further explore such relationships and hopefully follow up with mechanistic studies that will further clarify the relationships between specific 3’ end processing proteins and global changes in 3’ UTR length.*

Furthermore even for the positive controls, all patterns of correlation with 3'UTR ;ength changes (positive/negative/no correlation) are observed, depending on the cancer. Is there evidence that these patterns are so variable or do they represent study-specific biases?

*Reply: We thank the reviewer for this question. While it is true that variable patterns of correlation do occur for some of the positive controls, the most common outlier for those data is the colorectal adenocarcinoma dataset (e.g., as illustrated for CSTF2, CSTF2T, and PABPN1 in Supp. Figure 1A-C and shown in an excerpt from that figure copied below). We hypothesize that colorectal adenocarcinoma is an outlier for these analyses due to the fact that frequent APC mutations confound analyses of global correlations between 3’ UTR length and gene expression of known regulators. It is also possible that some of the observed variation reflects tissue-specific differences in poly(A) site selection (e.g., Aagarwal et al, Nature Communications, 2021).*

It appears that the authors found gene expression to be positively correlated with median 3'UTR, which is opposite to the general expectation. Can the authors explain this discrepancy?

*Reply: We thank the reviewer for this important question. The answer lies in a subtle distinction. As the reviewer notes, expression of a given gene X is typically expected to be negatively correlated with 3' UTR length of that gene X. However, we are performing a different comparison, where we correlate expression of a given gene X with the median 3' UTR length of all genes (not just the 3' UTR length of gene X). For a specific example, we might expect the expression of PABPN1 to be negatively correlated with the 3' UTR length of PABPN1. In contrast, the expression of PABPN1 is positively correlated with the median 3' UTR length computed across all genes (Supp. Figure 1C), suggesting that increased PABPN1 expression promotes use of longer 3' UTRs across the transcriptome – a finding that is consistent with previous mechanistic reports that high levels of PABPN1 reduce proximal poly(A) site selection (Jenal et al., 2012; Li et al., 2015).*

**Second round of review**

**Reviewer 1**

Thank you for the response to my questions and the questions posed by the other reviewers.
I have one additional question: The authors believe that the reason that the colorectal cancers do not show the same APA patterns as the other tumor types (Fig 1E, Supp Fig 1) is because they have APC mutations. The authors know which colorectal cancers have APC mutations. Why not look at colorectal tumors without APC mutations and report if only those colorectal tumors have similar patterns to the other tumors? Have the authors done this? I could not find it in the manuscript but perhaps I missed it. It's clear the colorectal cancer has a different APA pattern than other tumor types and that APC has a modest effect on APA, but are APC's effects sufficient to explain the striking difference in colorectal cancer APA?

**Reviewer 2**

I suggested that the authors analyze APC CLIP data, but their results were not satisfactory. All they showed was that APC binds near poly(A) signals. Does the binding pattern differ between proximal and distal polyA sites? Are the differences consistent with the APA changes? These questions were not addressed.

**Authors Response**

**Point-by-point responses to the reviewers’ comments:**

**Reviewer #1:**

Thank you for the response to my questions and the questions posed by the other reviewers.

I have one additional question: The authors believe that the reason that the colorectal cancers do not show the same APA patterns as the other tumor types (Fig 1E, Supp Fig 1) is because they have APC mutations. The authors know which colorectal cancers have APC mutations. Why not look at colorectal tumors without APC mutations and report if only those colorectal tumors have similar patterns to the other tumors? Have the authors done this? I could not find it in the manuscript but perhaps I missed it. It's clear the colorectal cancer has a different APA pattern than other tumor types and that APC has a modest effect on APA, but are APC's effects sufficient to explain the striking difference in colorectal cancer APA?

*Reply: We thank the reviewer for this comment. When we assess APC in comparison to other known factors that affect APA patterns globally, we see that APC expression is very strongly correlated with global poly(A) site use across all TCGA RNA-seq samples (Supp. Figure 1D, below).*

***APC is strongly correlated with global median 3’ UTR.*** *Waterfall plot of the mean Pearson correlation of gene expression (tpm) and median 3’ UTR length per sample across 30 TCGA cancer subtypes. Dotted lines indicate 5^th^ and 95^th^ percentile of Pearson correlations. Here a positive value indicates higher gene expression of the indicated gene is assoicated with, on average, longer 3’ UTRs. A more negative value indicates higher gene expression is associated with, on average, shorter 3’ UTRs. Blue dots are genes previously implicated in 3’ end processing, pink indicates APC and grey indicates all other genes.*

*The reviewer’s suggestion to stratify colorectal adenocarcinoma samples by their APC mutation status is insightful but also a challenging analysis to conduct, as even samples lacking an APC nonsense mutation or copy number deletion that was detected by whole-exome sequencing often harbor point mutations or other abnormalities that reduce levels of functional APC mRNA or protein or compromise APC function in unknown ways. Bearing this caveat in mind, we stratified samples by detected APC mutation status and found the global correlations between* *gene expression and median 3’ UTR were still similar between the colorectal adenocarcinoma samples) even after this stratification.*

***Similarity of TCGA cancer subtypes and gene expression – UTR correlations****. Heatmap of correlations of all datasets within TCGA compared to colorectal adenocarcinoma (COAD) data separated as*

*COAD (all samples), COAD.wt (only samples harboring no detected missense or nonsense mutations), COAD.mis (only samples harboring only missense mutations and no nonsense mutations), or COAD.mut (only samples harboring at least one nonsense mutation). Boxes colored by Pearson correlation coefficient (R).*

*Our observation of similar patterns for samples with different mutational status could arise from several causes. First, as the reviewer suggests, APC mutation status only partially explains the distinct pattern of APA in colorectal adenocarcinoma we observe. We agree with this conclusion; APC mutations are associated with clear changes in APA but are unlikely to be solely responsible. Second, many colorectal adenocarcinoma samples within the TCGA dataset that are annotated as having wild-type APC may still have APC that is subject to unusual mutations or other dysregulation. Recent long-read sequencing studies have shown that for many genes, including APC, large structural variants, inversions, and gene fusions can be missed via whole-exome, short-read sequencing methods used for the bulk of TCGA mutation analysis (Xu et al, PLOS Genetics 2023). Additionally, mutations in introns and other non-coding variants are known to disrupt gene function in familial adenomatous polyposis (Bozsik et al., European Journal of Human Genetics 2023), but these mutations are frequently considered of unknown significance unless they affect splice site dinucleotides. This would be in line with current understanding of the origin of colon adenocarcinoma, where abrogation of APC gene function is a key step in initiation of tumor formation (Zhang & Shay, J Natl Cancer Inst 2017). Regardless, further work will be needed to quantify and fully understand the extent to which APC mutations explain the distinct patterns of poly(A) site selection that we observe in colon adenocarcinoma.*

**Reviewer #2:**

I suggested that the authors analyze APC CLIP data, but their results were not satisfactory. All they showed was that APC binds near poly(A) signals. Does the binding pattern differ between proximal and distal polyA sites? Are the differences consistent with the APA changes? These questions were not addressed.

*Reply: We thank the reviewer for the comment and have done additional work in this revision to further elucidate the relationship between changes in 3’ UTR length and APA. Specifically, we complemented the previous analysis, which solely focused on canonical poly(A) sites, with analysis of the distance between identified APC binding sites and proximal versus distal poly(A) sites in 3' UTRs that were subject to APA. This new analysis revealed that APC binding was, on average, significantly closer to annotated, high-confidence poly(A) signal sequences that are the most proximal (first poly(A) site within the 3’ UTR) or intermediate (not the most proximal or most distal poly(A) site) than APC binding was to distal poly(A) sites (****Figure 4C-D, below****).*

***APC binds more closely to proximal poly(A) sites than it does to distal sites****. (****Left****) Example read coverage plots of APC HITS-CLIP binding data (Preitner et al 2013, Cell) for Sox11 and Rab23, demonstrating proximal binding within the 3’ UTR. (****Right****) Violin plots of distance between canonical poly(A) sites, binned by location within the 3’ UTR, as annotated per PolyA-DB v3. P value from a two-sided Wilcoxon-Rank Sum Test. Here, a positive value indicates that the poly(A) signal is downstream (more 3’) of the APC binding site and a negative value indicates the opposite.*

*To assess the implications of those findings from mouse neurogenesis HITC-CLIP data for APCmutant human colorectal adenocarcinoma, we assessed changes in poly(A) site selection in human colorectal adenocarcinoma samples among genes identified as APC binding targets from the mouse HITS-CLIP data. Given that these genes are bound by APC in mouse, we reasoned they may also be bound in human and thereby might exhibit more extreme changes in poly(A) site selection than did unbound genes in the context of APC loss. As hypothesized, we found that genes bound to APC in mice exhibited significantly more drastic changes in poly(A) site selection in the human clinical data (****Figure 4E, below****). This is particularly striking as the HITSCLIP data was conducted in mouse neurogenesis, while this poly(A) site selection analysis was completed using human colorectal adeocarcinoma data, suggesting a strong degree of conservation of APC RNA-binding between human and mouse.*

***APC binding targets display more dramatic 3’ UTR lengthening phenotypes****. Genes with significant changes in 3’ UTR length in human colorectal adenocarcinoma were grouped by whether or not they were identified as APC RNA-binding targets in the Preitner et al 2014 HITS-CLIP dataset. P value from a twosided Wilcoxon-Rank Sum*

*Test.*

**Third round of review**

**Reviewer 1**

Interesting study that I believe will be of interest to the broader community.

The additional data the authors provide are quite interesting and compelling that APC plays a role in APA selection. Thank you to the authors for performing the analysis that I requested and for sharing the results. Given that the colorectal tumors without detectable APC mutations (with the caveat that some may not be detectable) still cluster with other colorectal tumors, the authors may want to modify their discussion. Now they acknowledge that they don't know which APC-regulated APA changes are important for tumorigenesis. This is true. But the authors haven't shown that the only or even most important reason that colon cancer clusters differently from other tumors is the effects of APC. And their own data don't support it. But, a reasonable reader of this paper would draw that conclusion. The authors may want to add an acknowledgment of this in the discussion.

**Reviewer 2**

The authors have addressed my concerns sufficiently and therefore I support the publication of this manuscript.

**Authors Response**

**Point-by-point responses to the reviewers’ comments:**

**Reviewer #1:**

Interesting study that I believe will be of interest to the broader community.
The additional data the authors provide are quite interesting and compelling that APC plays a role in APA selection. Thank you to the authors for performing the analysis that I requested and for sharing the results. Given that the colorectal tumors without detectable APC mutations (with the caveat that some may not be detectable) still cluster with other colorectal tumors, the authors may want to modify their discussion. Now they acknowledge that they don't know which APC-regulated APA changes are important for tumorigenesis. This is true. But the authors haven't shown that the only or even most important reason that colon cancer clusters differently from other tumors is the effects of APC. And their own data don't support it. But, a reasonable reader of this paper would draw that conclusion. The authors may want to add an acknowledgment of this in the discussion.
 *Reply: We thank the reviewer for their comments and insightful discussion of our results. We agree with the reviewer that our data clearly demonstrate that APC plays a role in APA but do not prove that APC mutations are solely or even primarily responsible for the distinct patterns of APA that we observed for colon cancer. We revised the Discussion to clearly state this as suggested by the reviewer. In particular, we added this text:

“Given this, we cannot say with certainty that APC mutations are solely or even primarily responsible for the distinct global patterns of APA that we observed in colorectal adenocarcinoma samples (Figure 1E-F).”*

**Reviewer #2:**

the authors have addressed my concerns sufficiently and therefore I support the publication of this manuscript.
*Reply: We thank the reviewer for their time and comments. We believe their feedback has greatly improved the paper.*
